# An account of the Speech-to-Song Illusion using Node Structure Theory

**DOI:** 10.1371/journal.pone.0198656

**Published:** 2018-06-08

**Authors:** Nichol Castro, Joshua M. Mendoza, Elizabeth C. Tampke, Michael S. Vitevitch

**Affiliations:** Spoken Language Laboratory, Department of Psychology, University of Kansas, Lawrence, Kansas, United States of America; The University of Chicago, UNITED STATES

## Abstract

In the Speech-to-Song Illusion, repetition of a spoken phrase results in it being perceived as if it were sung. Although a number of previous studies have examined which characteristics of the stimulus will produce the illusion, there is, until now, no description of the cognitive mechanism that underlies the illusion. We suggest that the processes found in Node Structure Theory that are used to explain normal language processing as well as other auditory illusions might also account for the Speech-to-Song Illusion. In six experiments we tested whether the satiation of lexical nodes, but continued priming of syllable nodes may lead to the Speech-to-Song Illusion. The results of these experiments provide evidence for the role of priming, activation, and satiation as described in Node Structure Theory as an explanation of the Speech-to-Song Illusion.

## Introduction

Perceptual illusions occur when our percept does not match what is actually in the environment. Most people are familiar with visual illusions, some of which date back to Aristotle [1–2]. There are also illusions that involve both the visual and auditory modalities, such as the ventriloquist illusion ([3]; *cf*., [4]), and the McGurk effect ([5]; see also [6]). Finally, there are a number of illusions that are purely auditory in nature, including the Verbal Transformation Effect (VTE) [7], phonemic restoration [8], and verbal satiation [9]. Perceptual illusions in various modalities play an important role in increasing our fundamental understanding of perception and cognition.

In the present set of studies, we examined the auditory illusion known as the Speech-to-Song Illusion, which is elicited by continuously repeating a spoken phrase, without changing the stimulus in any other way. After several repetitions of the spoken phrase, listeners report that the stimulus now sounds as if it is being sung instead of spoken. Although this illusion was a well-known technique used by musicians who made loops of magnetic recording tape (e.g., “It’s gonna rain” by Steve Reich, 1965), the earliest report of this illusion in the scientific literature appeared in [10]. Deutsch discovered the Speech-to-Song Illusion while making instructional recordings describing other musical illusions [11–12]. When the spoken phrase “sometimes behave so strangely” was played over and over, listeners indicated that the stimulus changed from sounding like speech to sounding like song [10].

Since the initial report [10], the Speech-to-Song Illusion has been replicated with English phrases other than “sometimes behave so strangely” [13]. The illusion has also been observed in other languages including German [14] and Mandarin [15], further demonstrating the robustness of the illusion.

In addition to behavioral studies of the Speech-to-Song Illusion, Tierney, et al. [13] observed neurophysiological activity that corresponded to experiencing the illusion. Participants in an fMRI machine listened to phrases that they reported as either sounding like speech or sounding like song after repetition, even though all phrases were spoken. Six regions associated with pitch processing, vocalization, and auditory-motor integration, were more activated when the stimuli were perceived as being song-like rather than speech-like: anterior superior temporal gyrus bilaterally, right midposterior superior temporal gyrus, right lateral precentral gyrus, middle temporal gyrus bilaterally, left supramarginal gyrus, and left inferior frontal gyrus. Not only were participants indicating a subjective experience of the Speech-to-Song Illusion, but the brain also showed a different pattern of activation that included both speech and music processing areas of the brain.

Despite observing the Speech-to-Song Illusion in other languages, observing neurological correlates of the illusion, and several studies examining which stimulus characteristics increase or decrease perception of the illusion [13, 16], there is no account of the cognitive mechanism that underlies the Speech-to-Song Illusion. The lack of an underlying account of the Speech-to-Song Illusion is unfortunate, because such an account has the potential to greatly increase our fundamental understanding of speech perception, music perception, and auditory processing more generally.

Rather than introduce a unique and idiosyncratic model with special mechanisms to account just for the Speech-to-Song Illusion we, in what follows, test whether Node Structure Theory [17] can provide an account of the Speech-to-Song Illusion. Node Structure Theory (NST) is a connectionist model similar to (but distinct from in several very important ways) other spreading activation theories that describes the processes of perception (e.g., speech perception) and action (e.g., language production); see [17] for a book-length treatise of this model. NST has been used to account for normal memory and language processing (e.g., word retrieval and production; [17]), dysfunctional processing (e.g., tip-of-the-tongue states; [18]), the detection of speech errors [19], and differences in processing due to aging (e.g., [20]) or to certain cognitive deficits (e.g., amnesic-patient H.M.; [21]).

NST has also been used to account for the auditory illusion known as the Verbal Transformation Effect [22]. After briefly describing the central tenets of NST (see [17] for a more complete description of the model), we describe below several similarities in the Verbal Transformation Effect and the Speech-to-Song Illusion that motivated us to test NST as an account of the Speech-to-Song Illusion. We were also motivated to test NST because it is a general model of perception and action, and the perception of speech and of music may take place via certain domain-general mechanisms [23]. Although NST seeks to account for many aspects of speech perception and production we focus in this paper on examining only the processes in NST that are relevant to an understanding of the perceptual experience of the Speech-to-Song Illusion.

**Fig 1:**
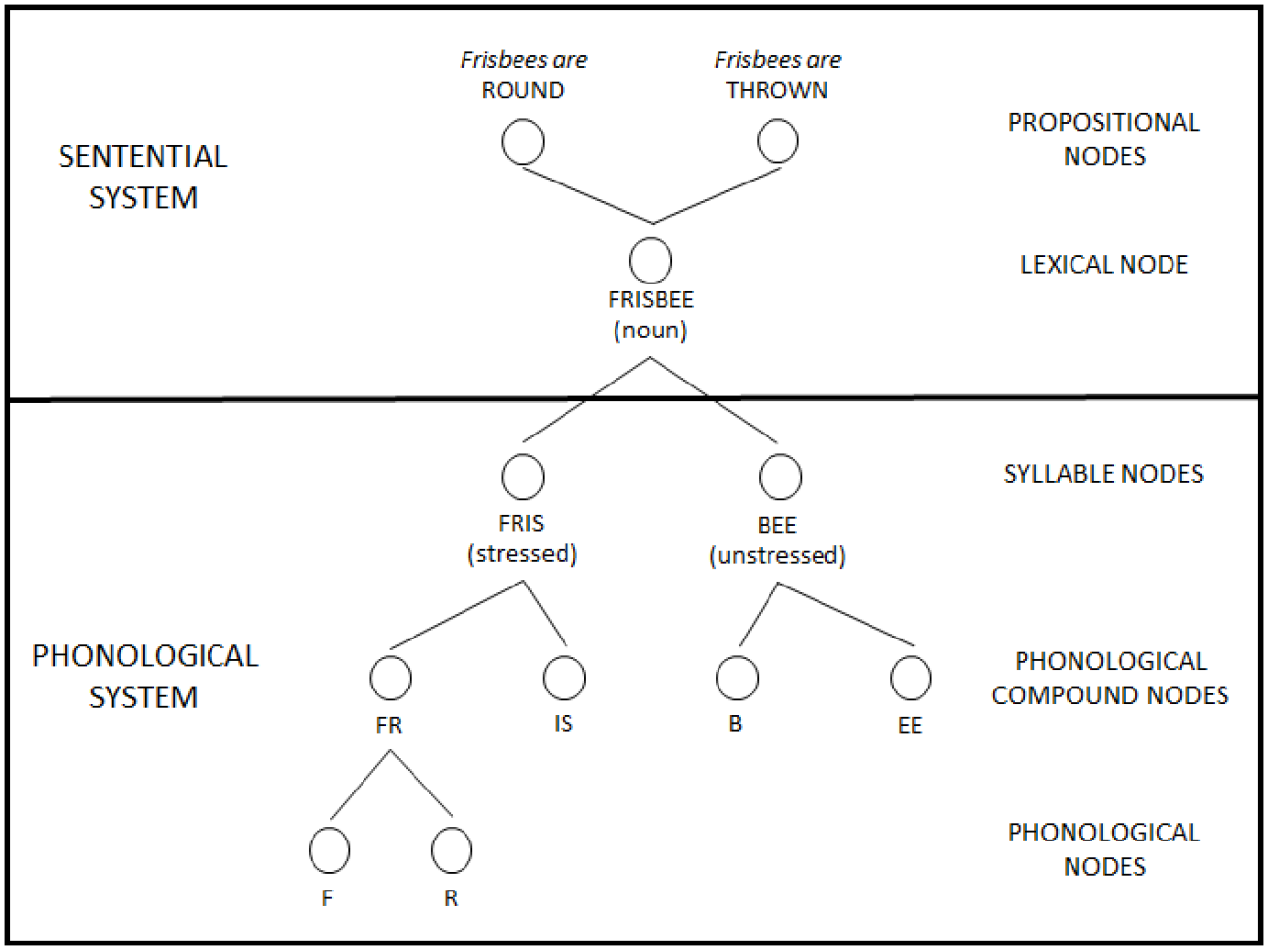
Depiction of the word *frisbee* according to Node Structure Theory. Adapted from Fig 1 in [17]. A simplified depiction of the network connections for the word *frisbee*, including higher-level sentential connections and lower-level phonological connections.

In NST, a node represents a piece of information, such as a phoneme, syllable, or word. Links connect related or constituent nodes together; phoneme nodes connect to syllable nodes, and syllable nodes connect to lexical nodes. The nodes are organized into different systems—such as the sentential system and the phonological system—with the nodes linked within and across systems [17]. As illustrated in Fig 1, the node for the word *frisbee* connects at the higher level to the semantic node “frisbees are thrown” and connects at lower levels to nodes for the syllables “fris” and “bee,” which in turn connect to the phonological nodes /f/, etc. Although not illustrated in Fig 1, the phonological nodes would eventually connect to motor nodes in order to articulate a word or phrase.

Although the representations employed in NST are similar to those used in many other spreading activation or connectionist models there is a crucial difference between NST and other spreading activation or connectionist models, namely the processes found in NST: priming, activation, and satiation. Priming increases activity in a node. During speech perception acoustic-phonetic input *primes* various phonological nodes, based on the extent to which nodes match the input. (The transmission of priming in NST might be referred to as “partial activation” in other models.) To become *activated* in NST, a node must accumulate enough priming summed across its connections (and over time) to surpass an activation threshold [17]. Activation of a node is “all-or-none,” and results in conscious awareness of the information represented by that node.

During speech production (and certain other processes) it is important to activate and properly sequence phonological units. Incorrect sequencing of activated nodes may result in the speech errors known as slips of the tongue. Failure to activate certain nodes may result in the inability to fully retrieve a word as occurs in the tip-of-the-tongue phenomenon. An important exception to the “all-or-none” principle of activation in NST is found in *speech perception* where it is typically sufficient to prime, but not fully activate units in the phonological system (including syllable nodes and nodes representing individual phonemes). The priming, but “failure” to fully activate syllable and phoneme nodes results in the listener perceiving words (which are activated) rather than sequences of phonemes when listening to speech [17].

Finally, repeated activation of the same node temporarily reduces the ability of that node to accumulate priming and be activated, leading to a state known as *satiation* [17]. Note that only nodes that have been activated experience satiation. MacKay, et al. [22] explicitly state that “[r]epeated priming may also cause satiation, but to a virtually negligible extent relative to repeated activation” (pg. 627). MacKay [17] further argues that the process of satiation serves the evolutionary function of bringing new stimuli to awareness instead of keeping old or unchanging information in awareness. When a given node is satiated, the “most-primed-wins” principle results in another related node being activated instead [17, 22].

The interplay among priming, activation, and satiation (as well as the “most-primed-wins” principle) has been used to account for a variety of findings in memory and language processing. Consider, for example, the work of MacKay, et al. [22] (see also [24–25]), which described how the processes of priming, activation, and satiation in NST account for the Verbal Transformation Effect (VTE; [7]), in which the percept of a repeated word changes into other words. For example, the word *base* played repeatedly to a listener might begin to be “heard” as *case*, or *face* because the lexical node for *base* becomes satiated, leaving the next “most-primed” lexical node (corresponding to *case* or *face*) to be activated and therefore perceived.

Given the change in percepts that occurs in both the Speech-to-Song Illusion and the VTE as a result of the stimulus being repeated, we reasoned that the same processes in NST—priming, activation, satiation—used to account for the VTE might also account for the Speech-to-Song Illusion (*N*.*B*., we return to the similarities between the VTE and the Speech-to-Song Illusion in the discussion of Experiment 6). Furthermore, priming, activation, and satiation are also used to account for normal language perception and normal language production, as well as the tip-of-the-tongue phenomenon, perceptual and motor adaptation, and the semantic satiation effect (another phenomenon in which stimulus repetition is involved). Given the breadth of phenomena accounted for by the processes of priming, activation, and satiation found in NST (and the similarity of certain phenomena such as the VTE, the semantic satiation effect and the Speech-to-Song Illusion) we sought to test whether NST could provide a parsimonious account of the cognitive mechanism that underlies the Speech-to-Song Illusion. Furthermore, there are not—at present, nor to our knowledge—any other accounts of the cognitive mechanism that underlies the Speech-to-Song Illusion, so we believed providing such an account could be informative about speech processing, music perception, and a number of other related areas of research.

Specifically, we reasoned that the repetition of the phrase in the Speech-to-Song Illusion causes the lexical nodes that correspond to the words in the phrase to satiate (i.e., they can no longer be activated), resulting in the loss of the initial “speech” percept. Importantly, however, despite satiation of the lexical nodes, the syllable nodes continue to be “stimulated” by the repeated phrase. In the terminology used in NST, the syllable nodes continue to receive priming, but they are not activated. Recall that priming *without* activation can and often does occur during everyday language perception, where one perceives words (because lexical nodes are activated), but one does not consciously perceive the sequences of syllables and phonemes that comprise the word (because nodes in the phonological level are only primed and not activated [17]).

With satiation of the lexical nodes, the “speech” percept is lost. However, the continued priming of the syllable nodes then leads to the emergence of the “song” percept in the Speech-to-Song Illusion. It is widely recognized that syllables are the unit of rhythmical structure in speech ([26]; *cf*., [27]), just as beats or notes serve as the unit of rhythmical structure in instrumental and vocal/choral music. Satiation of the lexical nodes, but continued priming of the syllable nodes, brings to conscious awareness the metrical pattern found in the repeated phrase, producing the song-like quality experienced by (many) listeners in the Speech-to-Song Illusion. The time-course we describe here—in which lexical nodes must be satiated, but the continued priming of syllable nodes then leads to the emergence of a song-like percept—is consistent with anecdotal reports of the phenomenology of the Speech-to-Song Illusion, where the “song” percept emerges after continued repetition of the phrase [28]. Furthermore, although repetition is a necessary component of the Speech-to-Song Illusion [28–30], Tierney and colleagues [28] argue that satiation of speech perception resources likely contributes to the illusion, but that there is no theoretical framework to explain how music-like qualities are extracted from a repeated spoken stimulus. Thus, the studies reported here test the mechanism we proposed to determine if NST can account for (at least certain aspects of) the Speech-to-Song Illusion.

## Experiment 1

Previous research on the Speech-to-Song Illusion used short phrases, such as “sometimes behave so strangely,” that were extracted from sentences [10]. Words spoken in a sentence are accompanied by an intonation contour that distinguishes statements from questions, and may focus attention on an important portion of the message (e.g., a disambiguating piece of information, such as, “*This* bag, not *that* bag.”). If repetition of the stimulus leads to satiation of the lexical nodes, but continues to prime the syllable nodes producing a metrical, music-like percept, then simply repeating words uttered independently and concatenated into a list should be sufficient to elicit the Speech-to-Song Illusion.

In addition, using words concatenated into a list allows us to strip away syntactic relationships that exist among words found in a phrase extracted from a sentence. Reducing syntactic influences is important in our test of the mechanisms in NST because a leading theory about the relationship between music and language suggests that a common syntactic processor might be used for both music and language [31]. If we are able to elicit the Speech-to-Song Illusion with a list of words rather than a phrase extracted from a sentence, then we will have also weakened a possible alternative hypothesis regarding the mechanism that is responsible for producing the Speech-to-Song Illusion. (To our knowledge, no one has explicitly stated that the *shared syntactic integration resource hypothesis* (SSIRH) proposed in [31] can account for the Speech-to-Song Illusion, but the SSIRH is a prominent hypothesis of the connections between language and music, so it is reasonable to consider this prominent model as a potential explanation for the Speech-to-Song Illusion, which is an illusion that involves both language and music percepts.)

We, of course, expect that a stimulus that differs from the original stimulus used to evoke the illusion (i.e., our stimulus lacks syntax and has reduced variability in intonation) is likely to result in a reduced effect of the Speech-to-Song Illusion. However, using such a stimulus will further generalize the illusion to other examples of speech. Furthermore, controlling variables such as syntax and intonation makes for a strong test of the mechanisms in NST that we propose underlie the Speech-to-Song Illusion.

To examine if the mechanisms in NST produce the Speech-to-Song Illusion we presented the word lists in three different conditions (across listeners): (1) just one presentation of each word list, (2) ten repetitions of each word-list with a 750ms pause between each repetition of the list, and (3) ten repetitions of each word-list with no pause between each repetition of the list. If repetition of the stimulus leads to satiation of the lexical nodes and a decrease in a speech-like percept, with continued priming of the syllable nodes leading to the emergence of the song-like percept, then the word list that is presented only once will not be sufficient to satiate the lexical nodes. We, therefore, predict that the list presented only once will be rated as more speech-like than the two conditions in which the lists are repeated.

In the repetition condition in which there is no pause between each repetition of the list, we predict that the lexical nodes that correspond to the words in the list will be satiated, causing the speech-like percept to be lost from conscious awareness. Because the syllable nodes are still receiving priming, we predict that the repetition condition in which there is no pause between each repetition of the list will bring to conscious awareness the metrical pattern found in the word-list, resulting in the stimuli in this condition to be rated as more song-like than the condition in which the list is presented only once.

Because the effects of satiation diminish with time in NST [17], in the repetition condition in which there is a pause of 750 ms before the list is repeated, we predict that the brief pause between repetitions may provide the lexical nodes with time to partially recover from the effects of satiation. Therefore, we predict that a song-like percept may be reported in this condition as well, but we expect that the ratings in this condition will fall between the ratings observed for the repetition condition in which there was no pause and the ratings for the lists presented only once.

Finally, if the processes in NST that account for normal language processing—as well as the tip-of-the-tongue state and the VTE—also account for the Speech-to-Song Illusion, then we would expect lexical characteristics that affect those other phenomena to also influence the Speech-to-Song Illusion. One lexical variable that has been shown to influence the tip-of-the-tongue state [32] and the VTE ([33]; see also [34–36]) is phonological neighborhood density, or the number of words that sound similar to a given word [37].

A word with a dense phonological neighborhood has many words that sound similar to it, whereas a word with a sparse phonological neighborhood has few words that sound similar to it. Phonological neighborhood density has not only been manipulated in experiments of the tip-of-the-tongue state and the VTE (two phenomena accounted for by NST), but it also has been manipulated in a large number of studies investigating speech perception, spoken word recognition, other aspects of speech production, word-learning, and various aspects of memory (for a review see [38]). Observing an effect of this well-studied variable in the present experiment would increase our confidence in the validity of any other effects that we might also observe. Therefore, instead of using 4 randomly selected words in each word-list in the present study, we constructed lists that contained 4 words that had dense phonological neighborhoods, or 4 words that had sparse phonological neighborhoods. Note that in both cases, none of the words in a given list were phonologically related to another word in that list.

In the VTE, words with dense phonological neighborhoods elicit more verbal transformations than words with sparse phonological neighborhoods ([33]; see also [34–36]). Given the similarity of the two phenomena (VTE and Speech-to-Song Illusion) we reasoned from the VTE results to predict that word-lists containing words with dense phonological neighborhoods would also be more susceptible to the Speech-to-Song Illusion, and would therefore be rated more song-like than lists containing words with sparse phonological neighborhoods.

In the context of NST we argue that the Speech-to-Song Illusion occurs because lexical nodes are initially primed and activated by the spoken input, giving the listener the percept of speech. With repetition of the stimulus the lexical nodes continue to be activated and eventually satiate, resulting in the loss of the speech percept. Although the lexical nodes have satiated the repetition of the stimulus continues to prime the syllable nodes. Given that syllables are the unit of rhythmical structure in speech, a more song-like percept then emerges.

Recall that syllable nodes receive priming but are typically not activated during normal speech perception, so they are not susceptible to satiation themselves. If they did succumb to satiation like the lexical nodes, then speech that contained alliteration or rhymes could not be perceived correctly. Such utterances would have the repeated phonemes or syllables “drop out” from the percept after several appearances in the utterance, significantly impairing the perception and comprehension of spoken language.

We further predicted, based on the findings of [33] with the VTE, that words with dense phonological neighborhoods would elicit higher song-like ratings than words with sparse phonological neighborhoods. In the context of NST lexical nodes for words with sparse phonological neighborhoods will be activated more quickly after recovering from satiation than lexical nodes for words with dense phonological neighborhoods. Because lexical nodes for words with sparse phonological neighborhoods become activated more quickly after recovering from satiation the speech percept for words with sparse phonological neighborhoods may be recovered for words with sparse phonological neighborhoods, thereby decreasing the song-like percept (and ratings) for such words.

To understand why lexical nodes for words with sparse phonological neighborhoods will be activated more quickly after recovering from satiation than lexical nodes for words with dense phonological neighborhoods consider the following example. The word *cat* has a dense phonological neighborhood (i.e., many words that sound like it). The phonological nodes of the constituent phonemes, /k/ /æ/ and /t/, not only transmit priming to the lexical node *cat*, but also transmit priming to phonologically similar words such as *bat*, *hat*, *can*, *cab*, *cut*, etc. In the case of a dense neighborhood the /k/ node for example must disperse its fixed amount of priming to many lexical nodes, resulting in each lexical node in a dense neighborhood receiving a smaller amount of priming compared to each lexical node in a sparse neighborhood where there are fewer similar sounding words that must share the priming being transmitted from the phoneme node.

If both a dense and sparse word experience satiation at time *t*, both words will recover from satiation at time *t*+1 (meaning they *can* be activated again *if* they receive sufficient amounts of priming). The sparse word could then be activated again at time *t*+2, because relatively more priming is being transmitted from the phonological nodes to the lexical node. However, because less priming is being transmitted from the phonological nodes to the lexical node in the case of a dense word (i.e., priming is being dispersed to more words), sufficient amounts of priming may not summate to activate the node until time *t*+3. With the lexical nodes for words with dense phonological neighborhoods not being activated for a longer period of time, the corresponding syllable nodes will instead continue to be primed producing the song-like percept for a longer period of time (and therefore higher song-like ratings).

### Method

#### Participants

In all of the experiments reported here we collected data until we obtained 30 participants (per condition) or the semester ended (resulting in some differences in sample sizes across the experiments reported here). All of the participants in the experiments reported here were undergraduate Psychology students enrolled at the University of Kansas who received partial course credit for their participation. Although age and other demographic information was not collected, none of the participants were minors. None of the participants reported any speech or hearing disorders, or participated in more than one experiment reported in this paper. All of the participants were native English speakers, and provided written informed consent before participating. Finally, all of the experiments reported here were approved by the institutional review board at the University of Kansas. In the present experiment, data were collected from ninety-five participants because five of the participants failed to follow the instructions of the task; their data were excluded from analyses (resulting in 30 participants per condition).

#### Materials

The 56 bisyllabic words with stress on the first syllable that were used in the present experiment were the same stimuli used in [39]. These items were recorded by a female, native English speaker at a normal speaking rate. Words were recorded in an IAC sound-attenuated booth using a high-quality microphone onto a digital recorder at a sampling rate of 44.1 kHz. The words were edited into individual sound files using Sound Edit 16 (Macromedia, Inc.).

The words were divided equally into two conditions: words with dense and words with sparse phonological neighborhoods. As reported in [39], dense words had a mean of 11.71 (*SD* = 1.58) phonologically similar words and sparse words had a mean of 4.43 (*SD* = 1.99) phonologically similar words. The difference between the two conditions was statistically significant, *F* (1, 54) = 229.88, *p* < .0001. Although the words differed in neighborhood density, they were controlled for word frequency, neighborhood frequency, phoneme length, and uniqueness points (all *p*s > .40), all had a strong-weak stress pattern, the same phonemes occurred in each condition equivalent numbers of times, and the number of fricatives that appeared in each condition was also equivalent. Furthermore, the durations of the words in the two conditions were also equivalent (dense words = 432 ms (SD = 49) and sparse words = 446 ms (SD = 44), *F* << 1).

The twenty-eight dense words were grouped into 7 lists, such that each list consisted of four words and each word was used only once. The twenty-eight sparse words were also grouped into 7 lists in a similar manner. None of the words in a list were phonological neighbors of another word in the list. Word-lists were matched between dense and sparse conditions in phoneme onset of each word (see word-lists in S1 Appendix). The minimum and maximum pitch values of the lists of words in the two conditions were equivalent (Minimum pitch for dense words = 161.60 Hz (SD = 53) and Minimum pitch for sparse words = 167.52 Hz (SD = 53); Maximum pitch for dense words = 309.82 Hz (SD = 109) and Maximum pitch for sparse words = 296.27 (SD = 80), all *p*s > .10).

Audacity 2.0.2 digital audio editor was used to concatenate the four separate sound files for each of the words into a single sound file/word-list. No additional time was included at the beginning or end of each sound file. Based on the duration values reported in [39], approximately 65 ms separated the end of one word from the beginning of the next word.

Participants listened to both the dense and sparse word-lists. As a between-subjects factor, there were three repetition conditions: no repetition, a 750 ms pause between repetitions, and repetition without a pause. In the no repetition condition, participants heard each word-list played once, as in [10]. In the repetition with pausing condition, participants heard the word-lists repeated 10 times with a 750 ms pause between each repetition. A final condition, not examined by [10], was the repetition without pausing condition. Participants heard each list repeated 10 times consecutively with no pause between repetitions. For the repetition conditions, Audacity 2.0.2 digital audio editor was used to produce a single sound file containing all the repetitions with or without pauses. There was no significant difference in total stimulus duration between the dense and sparse word-lists in each of the three repetition conditions (all *p*s > .25). Participants only participated in one of the three repetition conditions.

#### Procedure

Participants were tested in groups of up to three and seated individually at an iMac computer running PsyScope 1.2.2 [40]. Participants wore a set of Beyerdynamic DT 100 headphones and used a computer keyboard to indicate their ratings. PsyScope controlled stimulus presentation, played recordings to participants, and collected their responses.

Participants were asked to listen to and provide a rating on a 5-point Likert scale similar to that used in [10] with 1 corresponding to “sounds like speech” and 5 corresponding to “sounds like song.” Higher ratings on the scale indicate experiencing more of a song-like percept, whereas lower ratings on the scale indicate perceiving the stimulus as sounding more like normal speech.

Participants were prompted with the word READY for 500 ms on the screen to signal the start of a trial. After the list of four words was done playing (either once or 10 times depending on the condition), participants were prompted with “>” and typed in their rating using the computer keyboard. Participants then pressed the “return” key, which initiated the next trial. Participants heard each of the 14 stimuli (7 lists of dense words and 7 lists of sparse words) in a randomized order.

### Results

**Fig 2:**
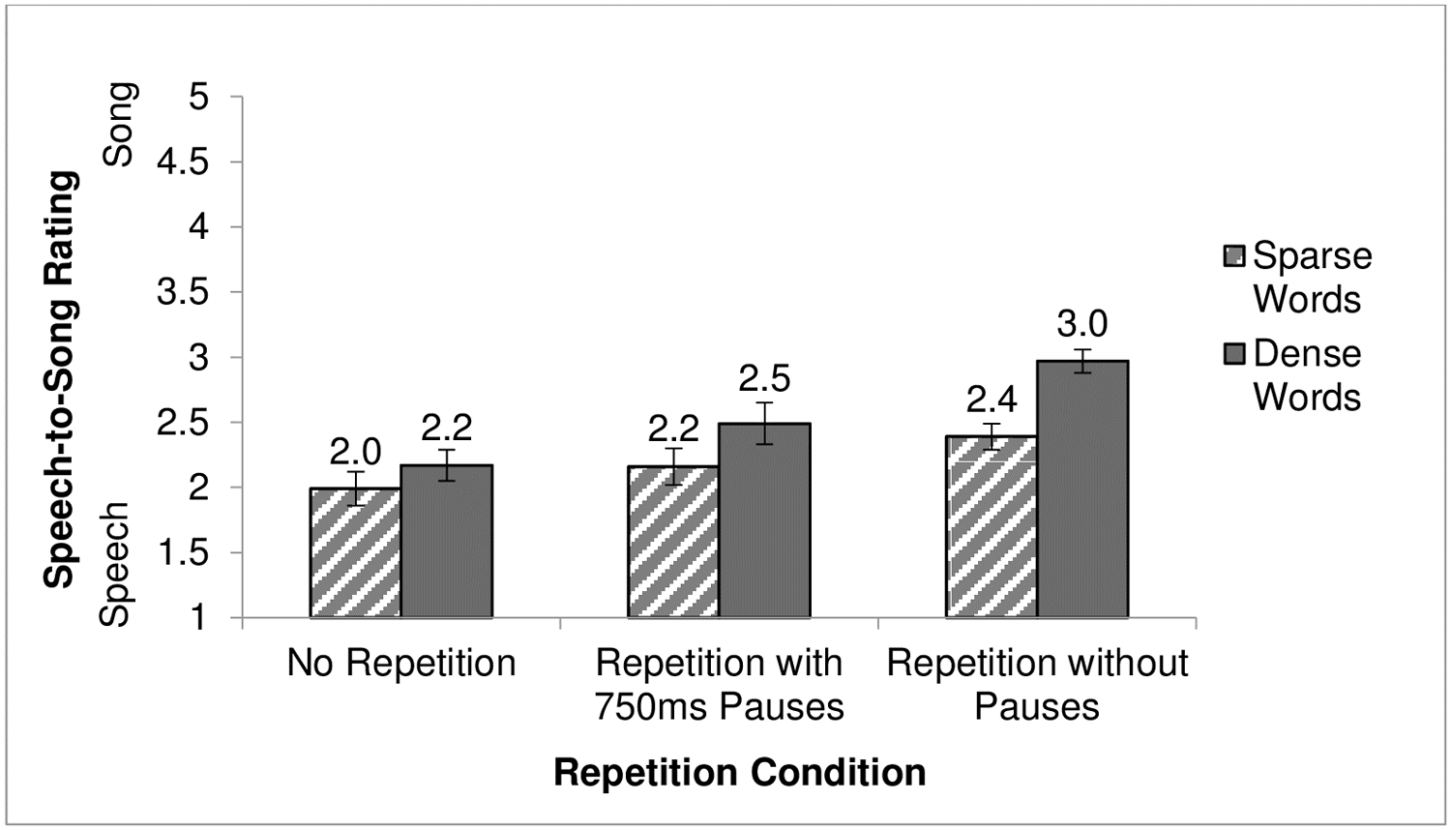
Speech-to-Song ratings of dense and sparse word-lists dependent on repetition condition. Ratings were made on a scale from 1 (sounds like speech) to 5 (sounds like song) for dense and sparse word-lists in three repetition conditions (no repetition, repetition with pauses, and repetition without pauses). Mean ratings for each condition are listed above each column, with whiskers representing standard error of the mean.

Participant ratings were analyzed in a 2 (density: dense and sparse) x 3 (repetition condition: no repetition, repetition with pause, and repetition with no pause) mixed ANOVA (see S1 Data). Phonological neighborhood density was a within-subjects condition and repetition condition was a between-subjects condition. A significant interaction between phonological neighborhood density and repetition condition was found, *F* (2, 87) = 3.47, *p* < .05 (see Fig 2). The effect size for the interaction (*η*^*2*^) was .06, which is considered a small effect size according to [41] and a medium effect size according to [42]. Because main effects should not be interpreted directly in the context of a significant interaction, simple main effects were instead calculated (using Bonferroni correction to adjust for multiple tests) to further explore how phonological neighborhood density and repetition condition influenced the Speech-to-Song Illusion.

The simple effect of repetition condition was examined at each level of phonological neighborhood density. For dense words, the repetition without pauses condition was significantly different from both the no repetition condition, *t*(87) = 4.42, *p* < .001, and the repetition with pauses condition, *t*(87) = 2.62, *p* < .05. However, the no repetition condition was not significantly different from the repetition with pauses condition, *t*(87) = 1.80, *p* = .23. Repetition without pauses elicited the strongest song-like percepts with dense word-lists.

For sparse words, the no repetition condition was not significantly different from either the repetition with pauses condition, *t*(87) = 0.96, *p* = 1.00, or the repetition without pauses condition, *t*(87) = 2.30, *p* = .07. Also, the repetition with pauses and the repetition without pauses conditions were not significantly different, *t*(87) = 1.34, *p* = .55. There was no difference in Speech-to-Song ratings amongst the repetition conditions for sparse words.

The simple effect of phonological neighborhood density was examined at each level of repetition condition (using a Bonferroni correction for multiple tests). For the no repetition condition, the mean difference between the average rating for dense word-lists (*M* = 2.17, *SD* = 0.68) and the average rating for sparse word-lists (*M* = 1.99, *SD* = 0.71) was not significant, *t*(87) = 1.74, *p* = .09. For the repetition with pauses condition, the mean difference between the average rating for dense word-lists (*M* = 2.50, *SD* = 0.86) and the average rating for sparse word-lists (*M* = 2.16, *SD* = 0.74) was significant, *t*(87) = 3.25, *p* < .01. Also, for the repetition without pausing condition, the mean difference between the average rating for dense word-lists (*M* = 2.97, *SD* = 0.51) and the average rating for sparse word-lists (*M* = 2.39, *SD* = 0.55) was significant, *t*(87) = 5.54, *p* < .001. In the two repetition conditions, dense word-lists were rated as sounding more like song than sparse word-lists. Without repetition, phonological neighborhood density did not significantly impact Speech-to-Song ratings.

### Discussion

The results of the present experiment showed several important things. First, repetition of a phrase extracted from a sentence, like “sometimes behave so strangely” [10], is not necessary to elicit the Speech-to-Song Illusion. As demonstrated in the present experiment, a list composed of four words that contained minimal music-like aspects of speech (like sentence or phrasal intonation, syntax, etc.) is sufficient to elicit music-like ratings. The fact that such impoverished stimuli produced an increase in music-like ratings provides strong evidence that the processes in NST—priming, activation, and satiation—may account for (at least certain aspects of) the Speech-to-Song Illusion.

At present there is, to our knowledge, no other model or theory that attempts to explain how the Speech-to-Song Illusion occurs. The *shared syntactic integration resource hypothesis* (SSIRH) [31] is a prominent theory that proposes a connection between language and music via shared syntactic resources, but even this hypothesis has not explicitly accounted for the Speech-to-Song Illusion despite the obvious connections between language and music inherent in the illusion. Eliciting an increase in music-like ratings in lists of four words that lack any syntactic structure as used in the present experiment suggests that syntactic information may not be necessary to elicit the Speech-to-Song Illusion, and that the cognitive mechanisms responsible for the illusion may not be related to syntactic processing.

Regarding the increases in music-like ratings observed in the present study, the initial study of the Speech-to-Song Illusion by [10] observed ratings in Experiment 1 approaching 4 on a 5-point rating scale. The ratings in the present experiment did not exceed the value of 3 on a 5-point rating scale, which may lead one to question whether we successfully elicited the Speech-to-Song Illusion at all. We note that there are a few differences in the methodology employed in [10] and in the present study that may account for the overall lower ratings observed in the present case (in addition to the obvious difference that [10] used a stimulus extracted from a sentence, whereas we used words spoken individually and concatenated into a list): Deutsch, et al. [10] used musically trained participants, whereas we did not; participants in [10] rated the stimuli after each repetition, whereas we obtained only a single rating at the end of 10 repetitions.

We further note that although our “song” ratings were not as high as those observed in the original study of the Speech-to-Song Illusion by [10], the ratings obtained in the present study are comparable to the ratings reported in a recent study of the Speech-to-Song Illusion by [43] and in another by [44]. More importantly, in the present set of studies it is the difference in the ratings across conditions that is of interest and is what is informative about the underlying cognitive mechanism, not the absolute value of the ratings.

A second important aspect of the present results is that stimulus repetition—especially repetition without a pause—plays a key role in eliciting the Speech-to-Song Illusion. Participants that heard the word-list repeated 10 times without a pause rated it as sounding more song-like than participants who heard the word-list presented only once. However, the repetition condition with a 750 ms delay diminished the perception of the Speech-to-Song Illusion.

Although the role of repetition in eliciting the Speech-to-Song Illusion contributes further to previous investigations that have examined the stimulus characteristics that evoke the illusion (e.g., [13, 16, 28–30]), we emphasize the role of repetition in eliciting the Speech-to-Song Illusion because of the role it plays in Node Structure Theory (NST). Namely, in NST repetition of the stimulus leads to satiation of the lexical nodes. With time, satiated nodes recover, enabling them to again accumulate priming and be successfully activated. We suggest that the 750 ms delay in the present experiment was sufficient to allow the lexical nodes corresponding to the words in the word-lists to recover (at least partially) from satiation, thereby reducing the experience of the Speech-to-Song Illusion.

Last, and perhaps most interesting, words with dense phonological neighborhoods elicited higher song-like ratings than words with sparse phonological neighborhoods after repetition. Because of the amount of priming transmitted by phonological nodes to the lexical nodes, words with sparse phonological neighborhoods will be activated more quickly after recovering from satiation than lexical nodes for words with dense phonological neighborhoods, thereby decreasing the song-like percept (and ratings) for such words. The results of the present experiment were consistent with that prediction.

Related to this last point, we also observed that the Speech-to-Song ratings of dense and sparse word-lists did not differ in the “no repetition” condition. Without repetition, the lexical nodes for each word do not satiate, allowing for accurate word retrieval and the perception of the stimulus as being very “speech-like.” With repetition, however, even a simple list of words can elicit the Speech-to-Song Illusion, consistent with the predictions we derived from NST.

Most importantly, however, the results of the present experiment support the hypothesis that the processes found in NST—priming, activation, and satiation—may account for the Speech-to-Song Illusion. Repetition of the stimulus leads to satiation of the lexical nodes and a loss of the speech percept, and a brief pause between repetitions can diminish the illusion. Continued presentation of the stimulus still primes the syllable nodes. Given that syllables are the unit of rhythmical structure in speech, the continued priming of the syllable nodes results in a percept that is more musical, or song-like.

We note that the processes found in NST—priming, activation, and satiation—may be necessary for the Speech-to-Song Illusion to occur, but they may not be sufficient. The results of the present experiment suggest that a syntactically meaningful phrase is not required for the illusion. However, other aspects of the auditory signal may enhance, reduce, or contribute in some way to the experience of the Speech-to-Song Illusion. For example, the stimuli in the present experiment were not only impoverished syntactically, they also were impoverished in their pitch and prosody. Experiment 1 in [10] found that when the repeated stimulus phrase was transposed (but preserved the formant frequencies and overall pitch contour) the song-like ratings were reduced, suggesting that some aspect of pitch may contribute to the perception of the illusion.

Indeed, recent findings by [45] indicate that certain regions of the brain devoted to processing pitch-related information in speech overlap with but are functionally independent from regions that process other information in the speech signal such as phonetic content and speaker identity. Given the overlap in processing areas in the brain, it is perhaps not surprising that the stimuli used in the present experiment, which sought to minimize variability in pitch, may have led to a slightly reduced experience of the Speech-to-Song Illusion. Nevertheless, the repetition of the stimulus in the present experiment was enough to satiate the lexical nodes and reduce the speech percept, with continued repetition of the stimulus priming the syllable nodes and producing a percept that is more musical, or song-like.

## Experiment 2

The results of Experiment 1 provided preliminary evidence that the processes found in NST may account for certain aspects of the Speech-to-Song Illusion. To further examine whether the processes found in NST can account for the Speech-to-Song Illusion, we considered another aspect of the model and how it might interact with the processes of priming, activation, and satiation. In NST, priming is transmitted more efficiently across links that are used more often than across links that are used less often [17]; this is how NST accounts for the ubiquitous word frequency effects observed in speech perception and speech production.

In English, most bisyllabic words have stress on the first syllable (a strong-weak stress pattern; like the words used in Experiment 1), and fewer bisyllabic words have stress on the second syllable (a weak-strong stress pattern). Given this difference in the frequency with which strong-weak and weak-strong words occur in the language, one would, in the context of NST, expect that priming would be transmitted more efficiently across the links between nodes associated with strong-weak words than across the links between nodes associated with weak-strong words. Just as the differences in the transmission of priming resulted in a difference in the susceptibility of words with dense versus sparse phonological neighborhoods in Experiment 1, we predict in the present experiment that the efficiency with which priming is transmitted to words with a strong-weak versus a weak-strong stress pattern will also lead to a difference in the susceptibility of such words to the Speech-to-Song Illusion.

Specifically, the less efficient transmission of priming to words with the less common weak-strong stress pattern will be more susceptible to the Speech-to-Song Illusion than words with the more common strong-weak stress pattern. Lexical nodes corresponding to words with a strong-weak stress pattern will have priming transmitted to them more efficiently, enabling them to be activated more quickly after being satiated. The reactivation of the lexical node will restore the speech percept and diminish the song-like percept (as well as song-like ratings).

On the other hand, lexical nodes corresponding to words with a weak-strong stress pattern will have priming transmitted to them less efficiently, requiring additional time to summate priming and activate the lexical node once it has been satiated. With the lexical node not being activated, the syllable nodes continue to receive priming, reinforcing the song-like percept (and leading to higher song-like ratings). Therefore, repeated lists of words with a weak-strong stress pattern in the present experiment will be rated more song-like than repeated lists of words with a strong-weak stress pattern.

### Method

#### Participants

Twenty-five undergraduate Psychology students enrolled at the University of Kansas received partial course credit for their participation in this experiment. The data from all of the participants were included in the analyses.

#### Materials

Forty bisyllabic words were used in the present experiment. Half of the words had stress on the first syllable (strong-weak stress pattern) and half had stress on the second syllable (weak-strong stress pattern). These items were recorded by a female, native English speaker (the first author) at a normal speaking rate in an IAC sound-attenuated booth using a high-quality microphone onto a digital recorder at a sampling rate of 44.1 kHz. The words were edited into individual sound files using Sound Edit 16 (Macromedia, Inc.).

The words were divided equally into two conditions: words with strong-weak and words with weak-strong stress patterns. The strong-weak and weak-strong words were controlled for frequency of occurrence, neighborhood density, number of phonemes, and recorded stimulus duration (all *p*s > .06). The words were selected from those listed in [46]. As described by [46], the same vowel that occurred in the stressed syllable of the strong-weak words also occurred in the stressed syllable of the weak-strong words. In addition, as much of the remaining phonological information was matched across the two syllable conditions as best as possible (see word-lists in S2 Appendix).

The twenty strong-weak stress pattern words were grouped into 5 lists, such that each list consisted of four words and each word was used only once. The twenty words with the weak-strong stress pattern were also grouped into 4 lists in a similar manner. The minimum and maximum pitch values of the lists of words in the two conditions were equivalent (Minimum pitch for strong-weak words = 100.00 Hz (SD = 40) and Minimum pitch for weak-strong words = 105.90 Hz (SD = 39); Maximum pitch for strong-weak words = 313.88 Hz (SD = 56) and Maximum pitch for weak-strong words = 268.10 (SD = 21), all *p*s > .50). Audacity 2.0.2 digital audio editor was used to concatenate the four separate sound files for each of the words into a single sound file. No additional time was included at the beginning or end of each sound file in order to approximate a natural speaking rate. Participants listened to both the strong-weak and weak-strong word-lists.

#### Procedure

The same equipment used in Experiment 1 was used in the present experiment. The same basic procedure used in Experiment 1 was also used in the present experiment, with the following exception. Participants heard the 10 stimuli (5 lists of strong-weak words and 5 lists of weak-strong words) in a randomized order. Each list was repeated 10 times without a pause between repetitions, and participants made their rating after the tenth repetition.

### Results

A repeated-measures two-tailed *t*-test revealed a statistically significant difference, *t* (24) = 2.88, *p* = .008, between the mean ratings from the strong-weak (*M* = 2.14, *SD* = .62) and weak-strong conditions (*M* = 2.58, *SD* = .70). This difference had a Cohen’s *d* = .66, which is considered a medium effect [42]. As predicted, lists of words with the less common weak-strong stress pattern were rated as being more song-like than lists of words with the more common strong-weak stress pattern (see S2 Data).

### Discussion

In NST, priming is transmitted more efficiently across links that are used more often than across links that are used less often [17]. Given that most bisyllabic English words have stress on the first syllable (a strong-weak stress pattern), and fewer bisyllabic English words have stress on the second syllable (a weak-strong stress pattern) we reasoned that the less efficient transmission of priming to words with the less common weak-strong stress pattern would be more susceptible to the Speech-to-Song Illusion than words with the more common strong-weak stress pattern.

Recall that lexical nodes corresponding to words with a strong-weak stress pattern will have priming transmitted to them more efficiently, enabling them to summate more quickly sufficient amounts of priming to activate the lexical node after being satiated. The reactivation of the lexical nodes corresponding to words with a strong-weak stress pattern may restore the speech percept and diminish the song-like percept (and the song-like ratings).

In contrast lexical nodes corresponding to words with a weak-strong stress pattern will have priming transmitted to them less efficiently, resulting in more time being required to summate sufficient amounts of priming to activate the lexical node after being satiated. The additional time required to summate priming in the lexical node gives more time for the syllable nodes to receive priming, thereby reinforcing the song-like percept and leading to higher song-like ratings.

The results of the present experiment were consistent with that prediction, providing additional evidence that the processes of priming, activation, and satiation in NST might provide an account of the Speech-to-Song Illusion. Repetition of the stimulus leads to satiation of the lexical nodes and a loss of the speech percept. However, continued presentation of the stimulus still primes the syllable nodes (i.e., the rhythmical units of language), resulting in a song-like percept. As demonstrated in the present experiment, the efficiency with which priming is transmitted between nodes affects how quickly a node will be activated again after satiation, and the extent to which the Speech-to-Song Illusion is perceived.

## Experiment 3

To further examine how NST might account for the Speech-to-Song Illusion, we used in the present experiment a list containing four nonwords. If satiation of the lexical nodes and continued priming of the syllable nodes are responsible for eliciting the Speech-to-Song Illusion, then we expect that the repetition of nonwords—which, by definition, do not have unique lexical nodes to activate, and would therefore only prime syllable nodes—will still produce the Speech-to-Song Illusion. Experiment 3 therefore represents a stronger test than the previous two experiments of the processes described in NST as an account of the Speech-to-Song Illusion.

In addition to “removing” lexical nodes from the experience of the Speech-to-Song Illusion by using nonwords, we again sought to manipulate the extent to which priming is transmitted among nodes as in the previous experiment. Recall in NST that priming is transmitted more efficiently across links that are used more often than across links that are used less often. Therefore, to manipulate the extent to which priming is transmitted to syllable nodes we used nonwords that varied in phonotactic probability. It is important to note that in Experiment 2 we examined how the transmission of priming from (stressed or unstressed) syllable nodes to lexical nodes affected the illusion. In the present study we will examine how the transmission of priming from the phoneme nodes to the syllable nodes affects the illusion by using nonwords that varied in phonotactic probability.

Phonotactic probability refers to the frequency of phonological segments and sequences of segments that occur in English words [47]. A nonword comprised of segments and sequences of segments that occur frequently in the language is said to have high phonotactic probability, whereas a nonword comprised of segments and sequences of segments that occur less frequently in the language is said to have low phonotactic probability.

With the less efficient transmission of priming from less common segments and sequences of segments to syllable nodes representing the nonwords we reasoned that lists containing nonwords with low phonotactic probability would not prime syllable nodes as efficiently as lists containing nonwords with high phonotactic probability. With more priming occurring at the syllable nodes that correspond to nonwords with high phonotactic probability, we reasoned that lists containing nonwords with high phonotactic probability would be rated as being more song-like than lists containing nonwords with low phonotactic probability.

In the NST account of the Speech-to-Song Illusion, once the lexical node is satiated the continued priming of the syllable nodes leads to the emergence of the song percept. We predict therefore that the amount of priming transmitted from the phoneme nodes to the syllable nodes—manipulated in the present experiment by using nonwords varying in phonotactic probability—will influence the extent to which the song percept emerges. Specifically, the more efficient transmission of priming from phoneme nodes to syllable nodes for nonwords with high phonotactic probability will lead to higher song-like ratings compared to lists containing nonwords with low phonotactic probability which transmit priming less efficiently from phoneme nodes to syllable nodes.

### Method

#### Participants

Sixty undergraduate Psychology students enrolled at the University of Kansas received partial course credit for their participation in the present experiment. The data from all of the participants were included in the analysis.

#### Materials

Fifty-six nonwords (28 high and 28 low phonotactic probability) from [47] were used in this experiment. All of the nonwords were recorded by the last author (a male, native English speaker) at a normal speaking rate using the same equipment and edited with the same procedure as described in the previous experiments.

Two measures of phonotactic probability were computed according to [48]: the sum of the segments and the sum of the sequences of segments. The sum of the segments for nonwords with high phonotactic probability (*M* = .16, *SD* = .03) was significantly greater than the sum of the segments for nonwords with low phonotactic probability (*M* = .09, *SD* = .02), *F* (54) = 10.12, *p* < .0001. The sum of the sequences of segments for nonwords with high phonotactic probability (*M* = .007, *SD* = .005) was also significantly greater than the sum of the sequences of segments for nonwords with low phonotactic probability (*M* = .001, *SD* = .001), *t* (54) = 5.81, *p* < .0001.

All of the nonwords were 3 phonemes in length, with an equal number of nonwords in the high- and low-phonotactic probability lists with the same onset phoneme. The mean number of phonological neighbors for the nonwords (based on the addition, deletion or substitution of a single phoneme to form a real English word; [37]) was 13.55 words.

The twenty-eight nonwords with high phonotactic probability were grouped into seven lists, such that each list consisted of four nonwords, and each nonword was only used once. The twenty-eight nonwords with low phonotactic probability were also grouped into 7 lists in a similar manner. Nonword-lists were matched between conditions in the phoneme onset of each nonword (see nonword-lists in S3 Appendix). The minimum and maximum pitch values of the lists of words in the two conditions were equivalent (Minimum pitch for high phonotactic probability words = 86.28 Hz (SD = 2.61) and Minimum pitch for low phonotactic probability words = 84.29 Hz (SD = 2.62); Maximum pitch for high phonotactic probability words = 147.32 Hz (SD = 3.71) and Maximum pitch for low phonotactic probability words = 172.34 (SD = 97.42), all *p*s > .28).

Audacity 2.0.2 digital audio editor was used to concatenate the four separate sound files for each of the nonwords into a single sound file. No additional time was included at the beginning or end of each sound file. The duration of each list of nonwords was also equivalent between conditions (mean duration for high phonotactic probability word lists = 2.50 s (SD = .08) and mean duration for low phonotactic probability word lists = 2.62 s (SD = .06), *p* > .28). Participants listened to both types of nonword-lists.

There were only two repetition conditions in the present experiment: no repetition and repetition (with no pause). In the no repetition condition, participants heard each list once. In the repetition condition, participants heard each list repeated 10 times consecutively. Audacity 2.0.2 digital audio editor was used to combine the four nonwords into lists, and to produce a single sound file containing all the repetitions, if any. There was no significant difference in the total stimulus duration between nonword-lists across phonotactic probability or between each of the repetition conditions (all *p*s > .80). Participants only participated in one of the two repetition conditions.

#### Procedure

The same equipment and procedure used in the previous experiments was used in the present experiment. Participants heard each of the 14 stimuli (7 high phonotactic probability and 7 low phonotactic probability nonword-lists) in a randomized order.

### Results

**Fig 3:**
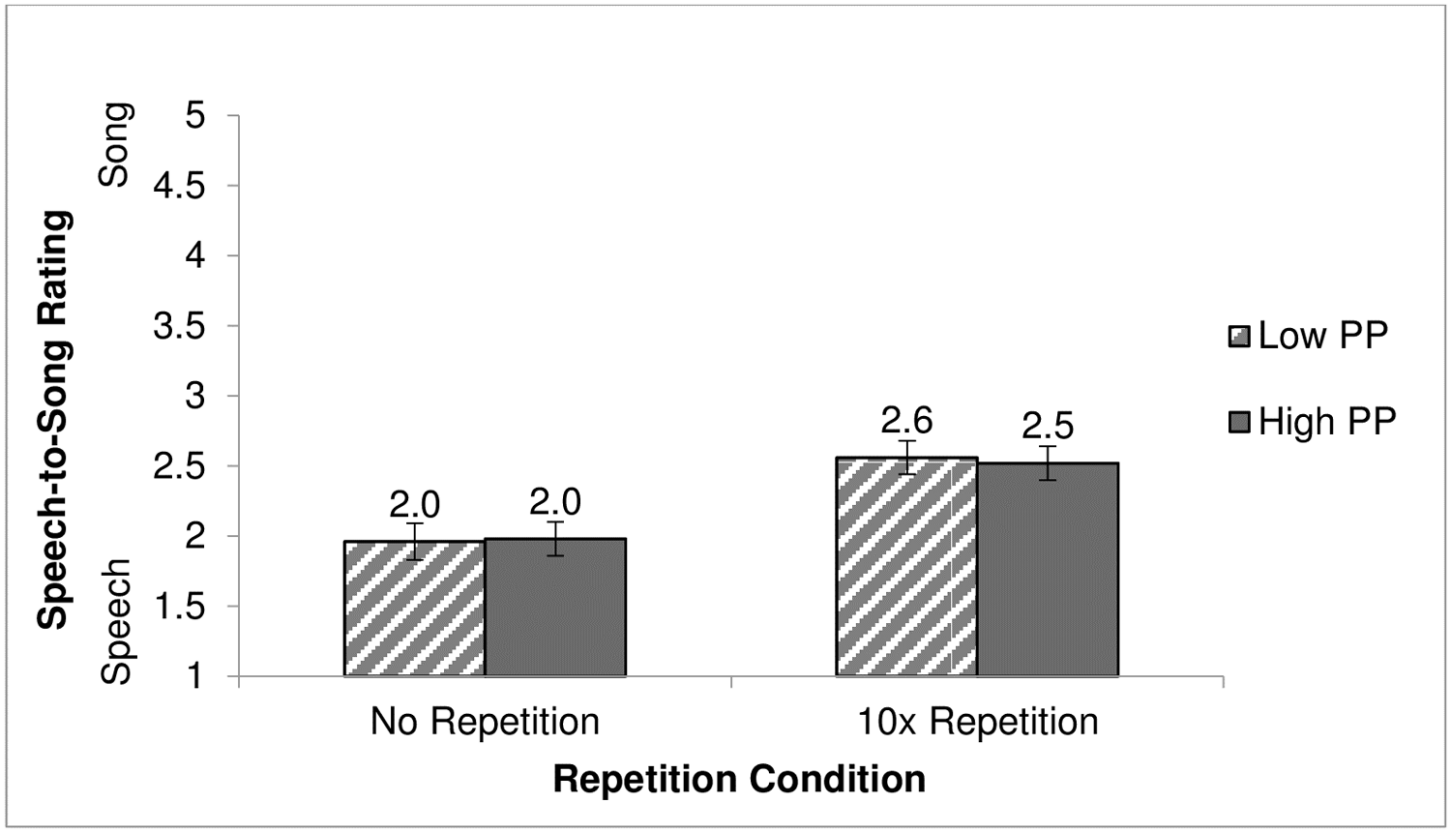
Speech-to-Song ratings of low and high phonotactic probability word-lists dependent on repetition condition. Ratings were made on a scale from 1 (sounds like speech) to 5 (sounds like song) for low and high phonotactic probability word-lists with and without repetition. Mean ratings for each condition are listed above each column, with whiskers representing standard error of the mean.

Participant ratings were analyzed in a 2 (phonotactic probability: low and high) x 2 (repetition condition: no repetition and 10 times repetition) mixed ANOVA (see S3 Data). Neither the interaction between phonotactic probability and repetition condition (*F* (1, 58) = 0.13, *p* > .05), nor the main effect of phonotactic probability (*F* (1, 58) = 0.10, *p* > .05) were significant. However, a main effect of repetition condition was significant, *F* (1, 58) = 13.36, *p* < .001 (see Fig 3). For the main effect of repetition condition, the effect size (*η*^*2*^) was .23, which is considered a large effect size according to [38] and [39]. Participants who heard the repeated stimuli rated the nonword-lists as more song-like than participants who heard the stimuli played only once.

### Discussion

In the present experiment we used lists of specially constructed nonwords that varied in phonotactic probability to test our hypotheses that the processes in NST could account for the Speech-to-Song Illusion. We reasoned that by using nonwords we would not need to satiate lexical nodes, because such nodes do not exist for nonwords. Instead, we could continually prime the syllable nodes—which play a role in the rhythmical structure of speech [26]—via repetition of the lists to produce a song-like percept. As we predicted, the repeated priming of the syllable nodes resulted in a higher song-like rating in the repeated lists of nonwords compared to the same lists of nonwords that were heard only once.

Somewhat surprisingly, phonotactic probability, which has been shown to influence a variety of language-related processes (e.g., [47, 49–50]) as well as other decisions (e.g., [51]), did not seem to influence the Speech-to-Song Illusion. We initially predicted that nonwords with high phonotactic probability would transmit priming more efficiently from the phoneme nodes to the syllable nodes than nonwords with low phonotactic probability. The more efficient transmission of priming would enable syllable nodes that correspond to the nonwords with high phonotactic probability to be more strongly primed, leading to higher song-like rating for nonwords with high rather than low phonotactic probability. This was not the case, however.

In an attempt to understand the failure to elicit an effect of phonotactic probability in this task we re-examined our nonword stimuli and discovered that 6 of the 30 nonwords with high phonotactic probability and 21 of the 30 nonwords with low phonotactic probability were CVC sequences that did not exist in English. That is, not all of our stimuli had extant syllable nodes. Having fewer stimuli than we thought contributing to the effects under investigation may explain why we did not observe the predicted influence of phonotactic probability in the present experiment.

Despite the failure to observe an influence of phonotactic probability on song-ratings in the present experiment, the results of the present study do provide some support for the processes we suggest underlie the Speech-to-Song Illusion. In NST we suggested that the repeated presentation of a list of words leads to satiation of the corresponding lexical nodes, but continued priming of the syllable nodes (i.e., the rhythmical units of speech). The satiation of lexical nodes and the continued priming of constituent syllable nodes produces the perceptual shift from a speech-like percept to a song-like percept that is characteristic of the Speech-to-Song Illusion.

In the present experiment in which we used nonwords, we found that even with the role of lexical nodes significantly reduced—recall that nonwords do not have lexical nodes—repeated priming of the syllable nodes led to higher song-like ratings in the repeated list condition compared to the condition in which the list was presented only once. To further test the hypothesis that repeated priming of syllable nodes leads to increased song-like ratings once lexical nodes have been satiated or otherwise “removed,” we tried again in the next experiment to “remove” lexical nodes, this time by using words from another language.

## Experiment 4

In Experiment 3 we used English-like nonwords that varied in phonotactic probability in an attempt to focus on priming the syllable nodes (i.e., the rhythmical units of speech) to more strongly test how the processes of priming, activation, and satiation in NST might account for the Speech-to-Song Illusion. In the present experiment we took a different approach to prime only the syllable nodes by using words from a foreign language. Words from a foreign language do not have lexical nodes in listeners who do not know that language.

Importantly, the phonotactic characteristics of the two languages used in the present experiment—English and Spanish—are similar enough that English syllables will be primed by the Spanish words. Indeed, all of the CV syllables in our Spanish stimuli are legal CV syllables in English. That is, even though a lexical node does not exist for our Spanish words in our monolingual English speakers, syllable nodes do exist (*cf*., the nonwords used in Experiment 3).

Furthermore, it is unlikely that Spanish words will prime or activate English words in native-English speakers who do not know Spanish. One reason it is unlikely that Spanish words will activate English words comes from work by Ju and Luce [52], who had fluent bilingual Spanish-English speakers participate in a listening task using the visual-world eye-tracking paradigm.

The listeners in the experiment by Ju and Luce [52] heard Spanish words that were spoken in a Spanish-like manner (with Spanish appropriate voice-onset-time (VOT) in the utterance) or Spanish words that were spoken in an English-like manner (with English appropriate VOTs). Using head-mounted eye-tracking, Ju and Luce [52] observed that the listeners fixated interlingual distractors (i.e., irrelevant English words that was phonologically similar to the spoken Spanish word) only when they heard the Spanish words spoken with English VOTs. When the listeners heard Spanish words with Spanish VOTs they did not fixate the interlingual distractors. In other words, listeners used fine-grained acoustic-phonetic information to activate only words in the “correct” language, rather than rampantly activating potential lexical candidates in all of the languages they know. The work of [52] is germane to the present study because the native-English speakers in the present study will be able to use the fine-grained acoustic-phonetic information in the input to determine that they are not listening to English words, and therefore will not activate English words.

Even if the Spanish words in the present study did prime or activate English words in the lexicon of our monolingual English listeners, an analysis of English and Spanish words by Vitevitch [53] demonstrated that there is very little lexical overlap of Spanish words with English words, and vice-versa. That is, when the entire vocabularies of the two languages are compared, there are relatively few Spanish words that are phonologically similar to English words (and vice-versa; see also [54] for similar findings using other pairs of languages). Thus, very few (if any) lexical competitors in English would be primed or activated by the Spanish input. Indeed, the Spanish words used in the present experiment were phonologically similar to (*mean* =) 1.93 English words (*cf*., *mean* = 13.55 English words for the nonwords in Experiment 3). Therefore, by using words from a foreign language we could more effectively prime the syllable nodes without potentially activating lexical nodes.

In the present experiment participants heard the lists of four English words from Experiment 1 repeated 10 times with no pause in between repetitions and lists of four Spanish words repeated 10 times with no pause in between repetitions (the words were from [55]). If satiation of the lexical nodes, but continued priming of the syllable nodes are responsible for eliciting the Speech-to-Song Illusion, then we expect that the repetition of Spanish words—which do not have lexical nodes to activate in listeners who do not know Spanish, and would therefore only prime syllable nodes—would result in higher song-like ratings than the repeated lists of English words, which will activate lexical nodes and initially elicit a speech-like percept before the lexical nodes satiate. Given that the Spanish words used in the present study were produced by a native speaker of Spanish (with Spanish VOT, etc.) and given the small amount of phonological overlap between Spanish and English words [53], it is unlikely that the Spanish words would activate English lexical nodes.

### Method

#### Participants

Twenty-six undergraduate Psychology students enrolled at the University of Kansas received partial course credit for their participation in this experiment. None of the participants in this experiment reported knowledge of Spanish (e.g., none were previously or currently enrolled in Spanish language classes, did not have a family member who spoke Spanish, etc.). The data from all of the participants were included in the analyses.

#### Materials

Ten lists of 4 English words from Experiment 1 (5 lists of dense words and 5 lists of sparse words) were used again in this experiment. In addition, the Spanish words produced by the female speaker in [55] were used to create 10 lists of 4 Spanish words (see [55] for more details about the Spanish stimuli, and the lists of words in S4 Appendix). Like the English words, the Spanish words also contained two syllables.

It is not surprising that the minimum and maximum pitch values of the two different speakers would differ for the lists of words used in the present experiment. The minimum and maximum pitch values of the lists of words in the two conditions were equivalent (Minimum pitch for Spanish words = 123.53 Hz (SD = 30.21) and Minimum pitch for English words = 164.56 Hz (SD = 53.06); Maximum pitch for Spanish words = 262.47 Hz (SD = 7.81) and Maximum pitch for English words = 303.05 (SD = 94.37). Importantly in the present experiment, the difference in pitch for the Spanish speaker (138.94 Hz) was comparable to the difference in pitch for the English speaker (138. 49 Hz), ruling out the possibility that acoustic differences between stimuli might be driving any differences we observe between the lists of English and Spanish words.

#### Procedure

The same equipment used in the previous experiments was used in the present experiment. Participants heard each of the 20 stimuli (10 lists of English words and 10 lists of Spanish words) in a randomized order. Each list was presented 10 times, and participants were asked at the end of the last repetition to provide a rating.

### Results

A repeated-measures two-tailed *t*-test revealed a statistically significant difference (see S4 Data), *t* (25) = 9.92, *p* < .0001, between the mean ratings from the English words (*M* = 1.65, *SD* = .62) and Spanish words (*M* = 3.27, *SD* = .69). This difference had a Cohen’s *d* = 2.49, which is considered a large effect [42].

### Discussion

As predicted, lists of Spanish words, which primed syllable nodes (i.e., the rhythmical units of speech) in the non-Spanish speaking participants, but did not activate lexical nodes were rated as being more song-like than lists of English words. The result of the present experiment provides evidence in support of the hypothesis that the repeated priming of syllable nodes contributes to the song-like percept in the Speech-to-Song Illusion.

It is important to note, however, that the Speech-to-Song Illusion is not due solely to priming of the syllable nodes (see also our discussion of pitch in Experiment 1). Recall that lexical nodes must first be activated in NST in order to experience the “speech” percept. The stimulus must also be presented repeatedly in order to satiate the lexical node, thereby reducing the perception of the stimulus as speech. The repetition of the stimulus also continues to transmit priming to the syllable nodes, which typically plays a role in the rhythmic aspects of speech. However, in this case once the lexical nodes have satiated, the continued priming of the syllable nodes leads to the emergence of a percept that is more musical or song-like than speech-like. NST therefore provides a complete and parsimonious account of these aspects of the Speech-to-Song Illusion. If one of these components is missing it is unlikely that one will experience the complete illusion: a speech percept followed by the transformation to a song percept. Furthermore, the presence or absence of other acoustic features (e.g., variation in pitch) may also contribute to the experience of the illusion.

In the present experiment we presented Spanish words to monolingual-English speakers in order to *avoid* activating lexical nodes, and just transmit priming to the syllable nodes. Although the present result provides evidence to support the mechanism in NST contributing to the song percept in the Speech-to-Song Illusion, it is not clear if participants in the present experiment experienced the complete illusion or simply experienced a song-like percept when listening to the lists of Spanish words. Unfortunately, we simply asked for a speech-song rating after each list had been repeated and did not ask participants explicitly if they had experienced the complete transformation with each stimulus. Nevertheless, the result of this experiment complements the findings obtained in Experiment 1, which demonstrated that continuous repetition of the stimulus contributes to the satiation of lexical nodes. Together the results of these experiments provide evidence for the processes of priming, activation, and satiation in NST accounting for the Speech-to-Song Illusion.

The result of the present experiment also provides a partial replication of an experiment on the Speech-to-Song Illusion reported by Margulis, et al. [43], where it was found that repeated excerpts of speech from various languages (including Catalan, Portuguese, French, Croatian, Hindi, and Irish) were rated as being more song-like than repeated excerpts of speech from English. The experiment by Margulis, et al. [43] is worth further discussion.

Margulis, et al. [43, pg. 2] reasoned that “…part of what distinguishes attending to music from attending to speech is a *participatory stance* (emphasis added), where the listener begins to sing through a tune in her head while it is playing after she has heard it a few times…” To test this hypothesis about music processing they repeated excerpts of speech from languages that were rated as being “easy” for a native speaker of English to pronounce (e.g., Catalan and Portuguese), of medium difficulty for a native speaker of English to pronounce (e.g., French and Croatian), or “hard” for a native speaker of English to pronounce (e.g., Hindi and Irish).

They predicted that languages that were easier for a native speaker of English to pronounce would be more susceptible to the Speech-to-Song Illusion and be rated more song-like than languages that were more difficult for a native speaker of English to pronounce. Not only did they find that the non-English languages were rated as being more song-like than English, as we noted above, but they also found the opposite of what they predicted: more difficult to pronounce languages were rated more song-like than easier to pronounce languages. Although the results obtained by [43, see also 44] were the opposite of the prediction from the participatory stance hypothesis (see also [56]), the results obtained by [43] can be accounted for by the processes in NST.

Recall that Vitevitch [53] examined the number of words in English that could be considered phonological neighbors of words in Spanish. He found that less than 5% of the words in one language were phonologically similar to a word in the other language (based on the addition, deletion, or substitution of a phoneme in a word), making it unlikely that the Spanish words used in the present study would activate English lexical nodes, but quite likely that they would still prime syllable nodes (by virtue of the two languages having similar phoneme inventories, both allow consonant-vowel syllable structures, etc.), thereby evoking the Speech-to-Song Illusion in native-English listeners who do not know Spanish.

Marian, et al. [54] did a similar analysis of words in English, Dutch, German, Spanish, and French, and found that, depending on the languages being compared, about 1–8% of the words in one language had a phonologically similar word in the other language (using the same one-phoneme metric used in [53]). Given the minimal amount of overlap of words across a number of languages it is perhaps not surprising that [43] observed that non-English languages were rated as being more song-like than English (as we found in the present experiment), because those non-English languages would only prime syllable nodes and not activate lexical nodes at all.

More interesting is the set of languages examined by [43], [53], and [54]. The English language can be thought of as a close cousin to other Germanic languages (e.g., Dutch, German) and other Romance languages (e.g., Spanish, French, Catalan, and Portuguese), but a more distant cousin to languages in other branches of language taxonomies, like Irish, a Celtic language, Hindi, an Indo-Aryan language, and Croatian, a Balto-Slavic language. The analyses by [53] and [54] considered languages that were close cousins to English, yet found minimal overlap of phonological neighbors. Given the more distant linguistic relationship to English of the languages rated intuitively as being more difficult to pronounce (i.e., Hindi and Irish) in [43], the likelihood that such languages would activate English lexical nodes is even more unlikely, leaving only syllable nodes to be primed, and resulting in a stronger song-like percept for the languages that were rated as more difficult to pronounce and are more distant cousins of English. Thus, although the results of the various languages observed by [43] appeared to be inconsistent with the participatory stance hypothesis that they were testing, their results are consistent with the hypothesis we derived from the NST that we are presently testing.

## Experiment 5

In Experiment 1 we tested the hypothesis that the processes in NST—priming, activation and satiation—might lead to the Speech-to-Song Illusion by using a list of four words instead of the semantically and syntactically intact phrases more typically used to illicit the illusion. In Experiments 2–4 we examined in various ways the role that priming plays in NST and in eliciting the Speech-to-Song Illusion. To examine further the processes in NST—this time focusing on satiation, or more precisely, recovery from satiation—that contribute to the Speech-to-Song Illusion, we used in the present experiment word-lists that contained from one to ten words.

Recall that satiation occurs after continued repetition of a node, and the effects of satiation diminish with time [17]. Therefore, we predicted that Speech-to-Song ratings would be highest (more song-like) for lists with fewer words, because each word in the list would be continually activated, leading to satiation of each of those lexical nodes, but continued priming of the constituent syllable nodes, and to the Speech-to-Song Illusion. For lists with many words we predicted that Speech-to-Song ratings would be lower (i.e., more speech-like), because the activation of many intervening words would allow each individual lexical node to recover from satiation before being activated again. With each lexical node recovering from satiation and being activated again, the experience of the Speech-to-Song Illusion would be reduced for longer word-lists.

### Method

#### Participants

Twenty-nine undergraduate Psychology students enrolled at the University of Kansas received partial course credit for their participation in the present experiment. One participant failed to follow the instructions of the task; their data were excluded from analyses.

#### Materials

To maximize our chance of observing the Speech-to-Song Illusion in the present experiment, we used the 28 dense words from Experiment 1, because this was the condition from Experiment 1 that elicited the greatest Speech-to-Song ratings. The words were grouped into lists that varied in the number of words, from one word up to ten words per list (see word-lists in S5 Appendix). There were five lists at each length yielding a total of 50 stimuli. Due to the limited number of dense words, the words were repeated across lists. Each list of words was repeated 10 times with no pause (again, the condition from Experiment 1 that elicited the greatest Speech-to-Song ratings). Audacity 2.0.2 digital audio editor was used to combine the individual words into lists and repeat the stimuli to create a single sound file for each word-list.

#### Procedure

The same equipment and procedure used in Experiment 1 was used in the present experiment. Participants heard each of the 50 stimuli in a randomized order.

### Results

A linear regression was calculated to determine the relationship between the number of words per stimulus and Speech-to-Song rating (see S5 Data). The aggregated mean and standard deviation for each word-list length is listed in Table 1. A negative correlation between the number of words per list and participant ratings was found, *R* = -.77, *F* (1, 9) = 11.76, *p* < .01. Listeners perceived the stimuli as sounding more like song when there were fewer words per list, and more like speech when there were more words per list.

**Table 1:** Mean and standard deviations of Speech-to-Song ratings for each word-list length.

Number of Words per List	Speech-to-Song Rating	
	*M*	*SD*
1	2.50	1.22
2	2.84	1.04
3	2.78	0.86
4	2.41	0.86
5	2.34	0.79
6	2.26	0.81
7	2.20	0.78
8	2.45	0.82
9	2.13	0.80
10	2.18	0.77

### Discussion

Consistent with our prediction, lists with more words were rated as sounding more speech-like, and lists with fewer words were rated as sounding more song-like. Having more words in a list increased the amount of time between subsequent activations of each word (and its corresponding lexical node) in the list, allowing for each node to recover from satiation and be activated again (i.e., evoking a speech percept), leading to a reduction in the song-like experience of the stimulus. The result of the present experiment further supports our hypothesis that the satiation of lexical nodes and the continued priming of syllable nodes in NST may be the cognitive mechanism that in part underlies the Speech-to-Song Illusion.

## Experiment 6

In the previous experiments we examined how the interplay of priming, activation, and satiation in NST lead to the Speech-to-Song Illusion. In the present experiment we further examined the interplay of these processes in NST by using multi-syllabic words to manipulate in a different way the amount of priming a syllable node receives. By varying the number of syllables in the words, we can vary the amount of priming that the constituent syllable nodes receive. Increasing the number of syllables in each word in the list will result in a set amount of priming being distributed among more constituent nodes. The more constituent syllable nodes there are, the smaller the proportion of priming each syllable will receive. Syllable nodes that receive less priming will be less likely to evoke the song-like percept. Therefore, words with many syllables will be rated as less song-like than words with fewer syllables.

### Method

#### Participants

Thirty undergraduate Psychology students enrolled at the University of Kansas received partial course credit for their participation in this experiment. The data from all of the participants were included in the analyses.

#### Materials

Eighty bi-syllabic words and thirty quad-syllabic words were used in this experiment. The words were combined into eight lists that varied in the number of words and the number of syllables (see word-lists in S6 Appendix). For the bi-syllabic words, the lists consisted of 1 word (2 syllables), 2 words (4 syllables), 3 words (6 syllables), 4 words (8 syllables), and 6 words (12 syllables). For the quad-syllabic words, the lists consisted of 1 word (4 syllables), 2 words (8 syllables), and 3 words (12 syllables). There were 5 lists for each list-length, for a total of 40 stimuli. Each word list was repeated 10 times consecutively. All of the words were recorded by the first author (a female, native English speaker) at a normal speaking rate in an IAC sound-attenuated booth using a high-quality microphone onto a digital recorder at a sampling rate of 44.1 kHz. The words were edited into individual sound files using Sound Edit 16 (Macromedia, Inc.). Audacity 2.0.2 digital audio editor was used to concatenate the words into a single sound file containing all repetitions.

The bi-syllabic and quad-syllabic words were controlled for phonological density, word frequency, and word familiarity. All words had no phonological neighbors (as defined by the single-phoneme metric used in [34]). The log frequency for bi-syllabic words (*M* = 1.20, *SD* = 0.42) and quad-syllabic words (*M* = 1.26, *SD* = 0.44) did not differ, *t* (108) = -0.57, *p* = .57. Words were also rated on familiarity using a 7-point scale, with higher ratings indicating greater familiarity. Bi-syllabic (*M* = 6.93, *SD* = 0.16) and quad-syllabic (*M* = 6.88, *SD* = 0.30) words did not differ in word familiarity, *t* (108) = 0.96, *p* = .34.

#### Procedure

The same equipment and procedure used in Experiment 1 was used in the present experiment. Participants heard each of the 40 stimuli in a randomized order.

### Results

A cross-classified mixed model was used to analyze the effect of the number of words and the number of syllables per stimulus on Speech-to-Song ratings (see S6 Data). This particular type of multilevel modeling allows examination of both the variability between subjects, *j*, and the variability between words, *i*. But most importantly this type of analysis allows us to examine the influence of the number of words and the number of syllables per stimulus even though these two variables are not fully crossed as would be required in ANOVA. That is, we can predict responses to combinations of syllables and words that we did not actually test.

To create the model, the number of words was centered at 1 and the number of syllables was centered at 2 (the shortest stimulus consisted of 1 word with 2 syllables). The quadratic effect of words and syllables was also examined because visual inspection of the data in Experiment 5 suggested a point of inflection between one and four words.

A hierarchical model building procedure and deviance test was used to determine significance of each effect on participant ratings. The fixed, linear effects of the number of words, γ_10_, and the number of syllables were added, γ_20_, respectively. Both linear effects were significant, and the quadratic effects were then added. Only the quadratic effect of words, γ_30_, was significant; the quadratic effect of syllables was excluded from the model. Once determining these fixed effects, the random effects of words, u_1*i*_, syllables, u_2*i*_, and words-squared, u_3*i*_, were included in that order.

**Fig 4:**
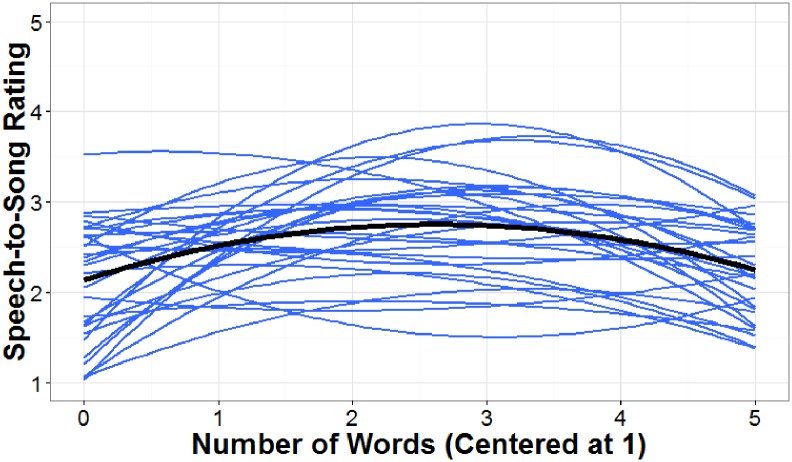
Plot of Speech-to-Song ratings dependent on the number of words per list. Spaghetti plot highlights the quadratic effect of the number of words on Speech-to-Song ratings for each participant. Blue lines represent each individual’s trajectory with the black line representing the average of all individuals.

**Fig 5:**
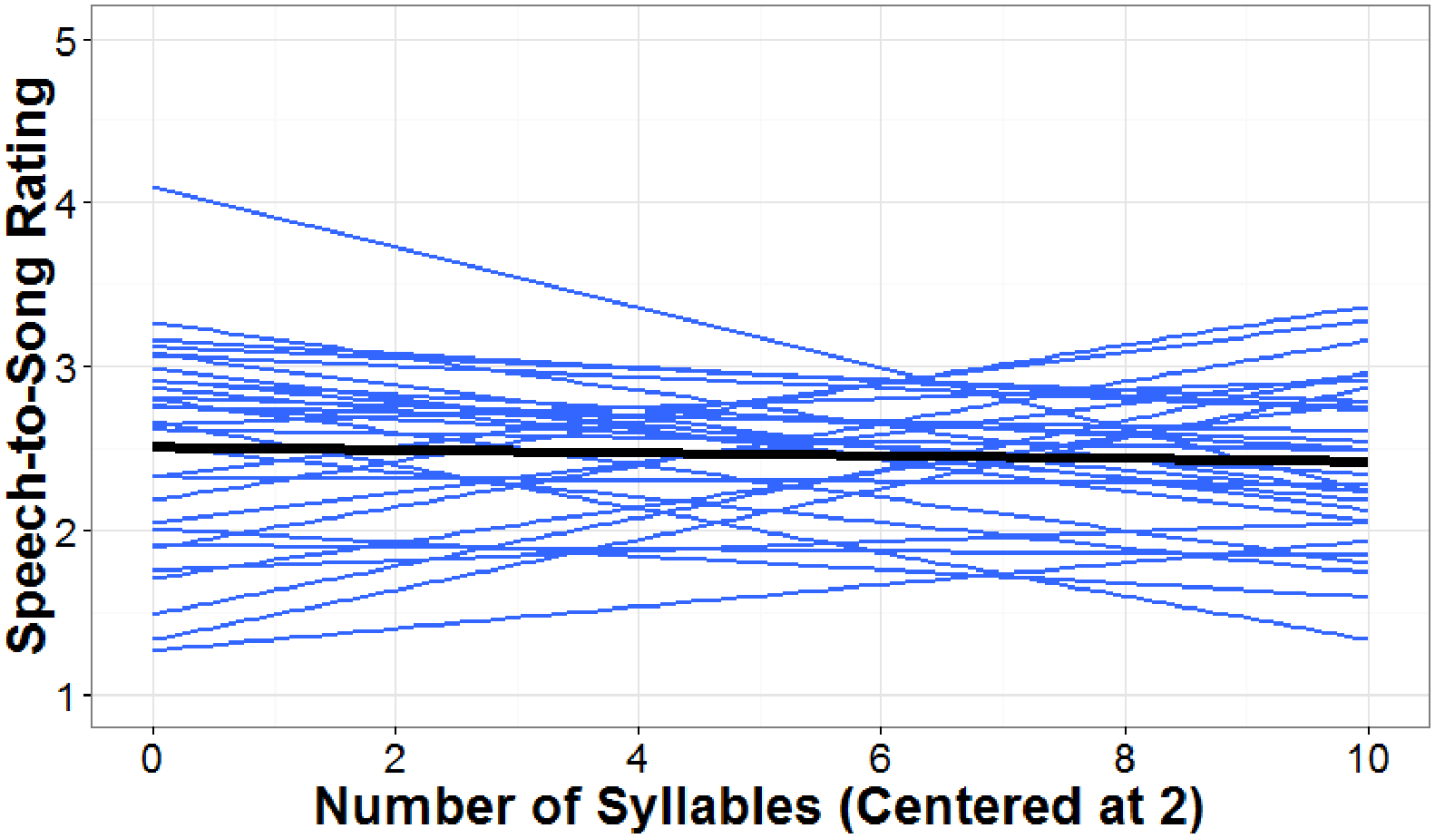
Plot of Speech-to-Song ratings dependent on the number of syllables per list. Spaghetti plot highlights the linear effect of the number of syllables on Speech-to-Song ratings for each participant. Blue lines represent each individual’s trajectory with the black line representing the average of all individuals.

All of the fixed effects were significant. A significant positive linear trend of the number of words (*t* = 6.64, *p* < .0001) and a significant negative quadratic trend of the number of words was found (*t* = -6.21, *p* < .0001). The linear trend shows that as the number of words increased by one (controlling for the number of syllables), the Speech-to-Song rating also increased by 0.79. However, the quadratic trend of -0.12 shows that as the number of words increased, the Speech-to-Song rating became more negative, and eventually became a negative slope (see Fig 4). There was also a significant negative linear trend of the number of syllables (*t* = -5.55, *p* < .0001), such that as the number of syllables increased by one (controlling for the number of words), the Speech-to-Song ratings also decreased by -0.11 (see Fig 5).

### Discussion

The general linear trend observed in the present experiment is consistent with the result of Experiment 5: increasing the number of words in the list decreased song-ratings. With a greater number of intervening words, a lexical node can recover from satiation, thereby decreasing the song-like percept.

Interestingly, the number of words per list also followed a quadratic curve in the present experiment, such that at approximately 3–4 words participants perceived the stimuli as sounding the most song-like. This novel finding suggests that an optimal number of words may be required to elicit the Speech-to-Song Illusion; too few or too many words will reduce the illusion. Although the goal of the present set of experiments was to investigate the processes in NST that may account for experiencing the Speech-to-Song Illusion—not to investigate which characteristics of the stimulus increase or decrease the illusion as previous studies have done [13, 16]—the results of the present experiment point to an interesting characteristic of the stimulus that may warrant future investigation: the number of words in the repeated phrase.

As we observed in Experiment 5, lists that contain more than 4 words allow lexical nodes to recover from satiation, leading to a decrease in song-like ratings. In addition to explaining why lists that contain more than 3–4 words sounded less song-like, NST may also account for why lists containing fewer than 3–4 words are also perceived as sounding less song-like. Recall that we initially appealed to NST because it had been used to account for the auditory illusion known as the Verbal Transformation Effect [57], in which the percept of a repeated word changes (e.g., the word *base* might be “heard” as *case*, or *face*). In the VTE one and sometimes two words are typically used as stimuli [57]. Perhaps with lists containing fewer than 3–4 words participants are more likely to experience verbal transformations rather than the illusory transformation from speech to song.

It is also interesting to note that Kaminska and Mayer [36] found during a VTE task that one of the non-identity changes described by participants was for word stimuli (one or two words in length, from one to four syllables in total) to be perceived as rhythmic. These interesting overlaps between the VTE and the Speech-to-Song Illusion suggest that there is much more to explore in these two illusions. Therefore, the results of the present set of experiments suggests that the mechanisms of priming, activation, and satiation found in NST may prove useful in understanding the Verbal Transformation Effect, the Speech-to-Song Illusion, and perhaps in understanding other auditory phenomena as well (e.g., [58]).

We also observed in the present experiment a negative linear trend for the number of syllables per list. When the number of words was controlled statistically, participants perceived the stimuli as less song-like as the number of syllables increased. Consider the illustrative example of two lists that each consist of 4 words, with the first list consisting of monosyllabic words, and the second list consisting of bisyllabic words; the list of monosyllabic words would be rated as sounding more song-like than the list of bisyllabic words.

NST can account for this novel finding as well. In the illustrative example of a list of bisyllabic words the priming that is transmitted to the syllable nodes will be disbursed or dissipated across more syllables, resulting in each syllable receiving less priming compared to the amount of priming that is transmitted to each syllable node in a list of monosyllabic words. With less priming being transmitted to each of the syllables in the illustrative example of a list of bisyllabic words, the song-like percept would be reduced for a list of bisyllabic words compared to a list with the same number of words that were monosyllabic. Because our stimuli ranged only from 2 to 12 syllables it is unclear at present if this negative linear trend will continue with stimuli that contain more than 12 syllables, or if some other trend (e.g., quadratic) might emerge. NST would predict the negative linear trend to continue with stimuli that contain more than 12 syllables. This clear prediction derived from NST can be easily falsified in future studies.

The results of the present experiment continue to point to the role of lexical nodes and syllable nodes as well as priming, activation, and satiation in the Speech-to-Song Illusion. The activation of lexical nodes produces the initial “speech” percept. Satiation of the lexical nodes, but continued priming of the syllable nodes—the structural units of rhythm in language ([26]—produce the “song” percept that emerges after a few repetitions of the phrase (or word-list as in the present experiments).

## General discussion

In the Speech-to-Song Illusion repetition of a spoken phrase produces a perceptual transformation resulting in spoken words sounding like they are being sung. The present study examined how the processes of priming, activation, and satiation in Node Structure Theory (NST; [17]) might provide the cognitive mechanism that underlies the illusion. The investigation of various auditory illusions to gain insight into the processing of music (e.g., [59]) and of language (e.g., duplex perception: [60]; sine wave speech: [61–62]) is not new. Similarly, the relationship between language and music processing is not new (e.g., [63–69]). For example, [70] found differences in the rhythm of children’s songs across cultures, perhaps related to differences in the speech rhythm of the languages examined (see also [71–72]). However, the present approach differs in a couple of important ways from previous research.

First, previous studies of the Speech-to-Song Illusion have typically manipulated certain characteristics of the stimulus to increase or decrease perception of the illusion ([13, 16]). The present work departs from this previous work in that we attempted to identify the cognitive mechanism that underlies the illusion (although some of our findings do have implications for the stimulus characteristics that increase or decrease the illusion). The results of the six experiments reported here suggest that the processes of priming, activation, and satiation in NST may account for certain aspects of the Speech-to-Song Illusion. Furthermore, our use of word-lists—which are devoid of the syntactic relationships found among words in a phrase—to elicit the Speech-to-Song Illusion casts doubt on a possible alternative mechanism for the illusion based on a syntactic processor common to both music and language [31].

In addition to describing the mechanism that may underlie the experience of the Speech-to-Song Illusion, NST might also be able to account for additional phenomena related to this illusion. For example, previous studies have observed that certain phrases are more effective at eliciting the illusion than others (e.g., [13]). As the results of Experiments 5 and 6 suggest, the number of words and syllables in the phrases (and the stress pattern of the words, as per the results of Experiment 2) may contribute to some phrases being more effective at eliciting the illusion than others.

As with other visual, audio-visual, and auditory illusions, not every individual experiences the Speech-to-Song Illusion. In the framework of NST variability in experiencing the illusion may be due to variability among individuals in the setting of various parameters, such that some people may require more priming than others, some may recover from satiation more quickly than others, etc. Perhaps some setting of these various parameters is optimal for experiencing the Speech-to-Song Illusion. Alternatively, perhaps there is an interaction between the parameter settings of individuals and the characteristics of the stimulus that is optimal for some listeners to perceive the illusion, but not optimal for another person [13, 16, 73]. In that case a slight change in the stimulus might make it less likely that the first individual experiences the illusion, but increases the likelihood that the second individual experiences the illusion.

One variation among individuals that has been well studied in the framework of NST is the efficiency with which priming is transmitted in older adults—the so-called transmission deficit hypothesis (e.g., [20])—where priming does not spread as efficiently in older adults as in younger adults. For example, the transmission deficit hypothesis is the leading account of why older adults experience the tip-of the-tongue phenomenon more than younger adults. If indeed the mechanisms found in NST underlie the Speech-to-Song Illusion, then one would predict that older adults are less likely to experience the Speech-to-Song Illusion than younger adults. Pilotti, et al. [74] found that older adults are less susceptible than younger adults to the verbal transformation effect, so our prediction regarding the Speech-to-Song Illusion is not unreasonable (and is readily falsifiable).

Our investigation of the Speech-to-Song Illusion suggests that Node Structure Theory might provide a way to bridge speech perception and music perception (e.g., [75]). Given the role that syllables play in the rhythmical structure of speech [26, 29; see also 45] and in the Speech-to-Song Illusion as the results of the present studies suggest, perhaps the bridge between speech perception and music perception lies at the syllable nodes rather than a common syntactic processor (*e*.*g*., [31]).

The use of Node Structure Theory (NST) to account for the Speech-to-Song Illusion leads to the intriguing question about the ability of NST to also account for other aspects of music processing, especially deficits in music processing. Recall that NST has been used to account for the cognitive deficits observed in amnesic patient, H.M. [21], and has also provided an account of the speech disorder of stuttering [76]. Perhaps NST can also provide insight into the music processing deficit known as amusia, especially in cases of amusia that involve the processing of rhythm. Further investigation is required to determine if NST can provide a parsimonious account of certain aspects of both language and music processing.

In addition to serving as a bridge between language and music processing the processes found in NST (and which may provide the cognitive mechanism for the Speech-to-Song Illusion) may also serve as a bridge between top-down and bottom-up influences on the processing of language and music. NST allows for top-down influences on auditory perception [17]. Indeed, recent work has shown that top-down processing, like musical knowledge [77] and attention [78], may be an important contribution to the experience of the Speech-to-Song Illusion. Furthermore, brain regions recruited for higher-level cognitive processing, specifically the frontotemporal loop, are activated when experiencing the illusion [29]. This evidence suggests that acoustic processing alone cannot account for the illusion, and further supports the effort here to provide a cognitive mechanism that underlies the Speech-to-Song Illusion. Additional investigation is required to assess the extent to which top-down and bottom-up factors influence the Speech-to-Song Illusion and whether NST can adequately account for those infleunces.

Although verbal models, like NST, are often criticized for not being specific enough to allow for clear predictions, we have put forward several specific predictions about the Speech-to-Song Illusion derived from NST that are easily falsifiable. We welcome tests of these predictions in the near future.

## Supporting information

S1 Appendix List of words used in Experiment 1. Each list contains 4 words that are all either dense words or sparse words. All word-lists are given.
(DOCX)

S2 Appendix List of words used in Experiment 2. Each list contains 4 words that all either have a strong-weak stress pattern or weak-strong stress pattern. All word-lists are given.
(DOCX)

S3 Appendix List of nonwords used in Experiment 3. Each list contains 4 nonwords that all either have high phonotactic probability or low phonotactic probability. All word-lists are given.
(DOCX)

S4 Appendix List of Spanish words used in Experiment 4. Each list contains 4 Spanish words. All word-lists are given.
(DOCX)

S5 Appendix Lists of words used in Experiment 5. Lists vary in the number of words from 1 word to 10 words. All word-lists are given.
(DOCX)

S6 Appendix Word-lists used in Experiment 6. Lists vary in the number of words and the number of syllables from 1 to 6 words and 2 to 12 syllables. All word-lists are given.
(DOCX)

S1 Data Summary data from Experiment 1. (CSV)

S2 Data Summary data from Experiment 2. (CSV)

S3 Data Summary data from Experiment 3. (CSV)

S4 Data Summary data from Experiment 4. (CSV)

S5 Data Summary data from Experiment 5. (CSV)

S6 Data Summary data from Experiment 6. (CSV)
