# Supplementary material for: An account of the Speech-to-Song Illusion using Node Structure Theory
Source: PLoS One. 2018 Jun 8;13(6):e0198656. doi: 10.1371/journal.pone.0198656 (PMC5993277; doi:10.1371/journal.pone.0198656)
Supplement: S1 Appendix — Each list contains 4 words that are all either dense words or sparse words. All word-lists are given. (DOCX) [file pone.0198656.s001.docx]

**S1 Appendix. List of words used in Experiment 1.** Each list contains 4 words that are all either dense words or sparse words. All word-lists are given.

| **Dense Word-Lists** | | | | **Sparse Word-Lists** | | | |
| --- | --- | --- | --- | --- | --- | --- | --- |
| lever | battle | furry | candle | lumber | badger | formal | cancer |
| letter | muscle | berry | babble | lawyer | mother | button | barrel |
| polar | bubble | money | ladder | person | beggar | movie | lucky |
| cattle | banner | tackle | hurry | cashew | burden | tower | hero |
| leather | valley | puddle | candy | lady | vapor | powder | camel |
| dairy | meter | body | lighter | devil | mighty | bottom | lotion |
| paddle | shallow | mayor | worry | purple | shower | mitten | water |
