# Supplementary material for: An account of the Speech-to-Song Illusion using Node Structure Theory
Source: PLoS One. 2018 Jun 8;13(6):e0198656. doi: 10.1371/journal.pone.0198656 (PMC5993277; doi:10.1371/journal.pone.0198656)
Supplement: S2 Appendix — Each list contains 4 words that all either have a strong-weak stress pattern or weak-strong stress pattern. All word-lists are given. (DOCX) [file pone.0198656.s002.docx]

**S2 Appendix. List of words used in Experiment 2.** Each list contains 4 words that all either have a strong-weak stress pattern or weak-strong stress pattern. All word-lists are given.

| **Strong-weak word lists** | **Weak-strong word lists** |
| --- | --- |
| gargle partner flutist lazy | regard depart pollute ablaze |
| final sinus manger cable | define assign remain decay |
| rover further tuna lighter | corrode refer attune polite |
| elder rainy servant feudal | repel arraign deserve refute |
| matron fated curdle porter | amaze inflate occur report |
