# Supplementary material for: An account of the Speech-to-Song Illusion using Node Structure Theory
Source: PLoS One. 2018 Jun 8;13(6):e0198656. doi: 10.1371/journal.pone.0198656 (PMC5993277; doi:10.1371/journal.pone.0198656)
Supplement: S3 Appendix — Each list contains 4 nonwords that all either have high phonotactic probability or low phonotactic probability. All word-lists are given. (DOCX) [file pone.0198656.s003.docx]

**S3 Appendix. List of nonwords used in Experiment 3.** Each list contains 4 nonwords that all either have high phonotactic probability or low phonotactic probability. All word-lists are given.

| **High PP** | | | | **Low PP** | | | |
| --- | --- | --- | --- | --- | --- | --- | --- |
| bæz | mɛk | faɪs | pɪm | bɔʄ | moɪk | fɑutʄ | pɝg |
| mos | tæs | dɛm | dʒoɹ | mɝz | teʄ | dɔb | dʒaɪg |
| gɛl | bɛs | nʌs | ɹɛn | gɝp | bɝʄ | nɑub | oɪk |
| ɹɛs | nɪd | gɪd | des | ɹɑub | nɝg | gɑub | deð |
| pɪz | dʒɪt | bɪθ | faɪd | pʊtʄ | dʒɝʄ | baɪdʒ | fɝp |
| tes | fɪm | mɑun | gaɪn | todʒ | foɪz | mɑub | gaɪθ |
| dʌp | paɪd | dʒæd | nɛs | dɝf | pudʒ | dʒeʄ | nɛʄ |
