# Supplementary material for: An account of the Speech-to-Song Illusion using Node Structure Theory
Source: PLoS One. 2018 Jun 8;13(6):e0198656. doi: 10.1371/journal.pone.0198656 (PMC5993277; doi:10.1371/journal.pone.0198656)
Supplement: S4 Appendix — Each list contains 4 Spanish words. All word-lists are given. (DOCX) [file pone.0198656.s004.docx]

**S4 Appendix.** **List of Spanish words used in Experiment 4.** Each list contains 4 Spanish words. All word-lists are given.

| duna fuga liga pena |
| --- |
| daga fama losa pata |
| gozo tubo foso lujo |
| gato tiro foro loro |
| lona nata roca tapa |
| lupa nuca ruta tela |
| mora lata sala rana |
| mina lona seda rima |
| rabo moho luto bano |
| rayo mito lago beso |
