# Supplementary material for: An account of the Speech-to-Song Illusion using Node Structure Theory
Source: PLoS One. 2018 Jun 8;13(6):e0198656. doi: 10.1371/journal.pone.0198656 (PMC5993277; doi:10.1371/journal.pone.0198656)
Supplement: S5 Appendix — Lists vary in the number of words from 1 word to 10 words. All word-lists are given. (DOCX) [file pone.0198656.s005.docx]

**S5 Appendix. Lists of words used in Experiment 5.** Lists vary in the number of words from 1 word to 10 words. All word-lists are given.

| **1 Word per List** | | | | | | | | | |
| --- | --- | --- | --- | --- | --- | --- | --- | --- | --- |
| cattle |  |  |  |  |  |  |  |  |  |
| candle |  |  |  |  |  |  |  |  |  |
| polar |  |  |  |  |  |  |  |  |  |
| banner |  |  |  |  |  |  |  |  |  |
| furry |  |  |  |  |  |  |  |  |  |
| **2 Words per List** | | | | | | | | | |
| ladder | money |  |  |  |  |  |  |  |  |
| worry | leather |  |  |  |  |  |  |  |  |
| hurry | meter |  |  |  |  |  |  |  |  |
| tackle | battle |  |  |  |  |  |  |  |  |
| babble | dairy |  |  |  |  |  |  |  |  |
| **3 Words per List** | | | | | | | | | |
| lever | shallow | paddle |  |  |  |  |  |  |  |
| dairy | babble | worry |  |  |  |  |  |  |  |
| bubble | lighter | money |  |  |  |  |  |  |  |
| ladder | valley | battle |  |  |  |  |  |  |  |
| cattle | banner | candy |  |  |  |  |  |  |  |
| **4 Words per List** | | | | | | | | | |
| furry | mayor | leather | tackle |  |  |  |  |  |  |
| polar | letter | candy | battle |  |  |  |  |  |  |
| lever | cattle | tackle | hurry |  |  |  |  |  |  |
| candle | banner | puddle | berry |  |  |  |  |  |  |
| hurry | lighter | babble | ladder |  |  |  |  |  |  |
| **5 Words per List** | | | | | | | | | |
| battle | muscle | polar | money | hurry |  |  |  |  |  |
| shallow | furry | puddle | lever | body |  |  |  |  |  |
| worry | mayor | babble | paddle | banner |  |  |  |  |  |
| lighter | candy | muscle | tackle | letter |  |  |  |  |  |
| berry | meter | leather | candle | bubble |  |  |  |  |  |
| **6 Words per List** | | | | | | | | | |
| worry | babble | letter | cattle | dairy | candy |  |  |  |  |
| paddle | berry | valley | mayor | letter | candle |  |  |  |  |
| money | puddle | leather | banner | furry | babble |  |  |  |  |
| bubble | lighter | body | lever | worry | puddle |  |  |  |  |
| shallow | tackle | battle | polar | leather | hurry |  |  |  |  |
| **7 Words per List** | | | | | | | | | |
| paddle | hurry | banner | ladder | valley | berry | mayor |  |  |  |
| babble | lighter | muscle | cattle | tackle | meter | hurry |  |  |  |
| cattle | battle | candy | lever | dairy | polar | candle |  |  |  |
| furry | leather | mayor | body | shallow | valley | bubble |  |  |  |
| letter | money | dairy | puddle | ladder | worry | paddle |  |  |  |
| **8 Words per List** | | | | | | | | | |
| leather | tackle | candy | polar | bubble | lever | meter | battle |  |  |
| battle | banner | paddle | ladder | shallow | dairy | money | cattle |  |  |
| letter | shallow | mayor | berry | muscle | valley | worry | lighter |  |  |
| mayor | paddle | babble | shallow | hurry | dairy | polar | money |  |  |
| candle | muscle | valley | furry | puddle | cattle | lever | meter |  |  |
| **9 Words per List** | | | | | | | | | |
| dairy | puddle | lighter | berry | bubble | body | candle | furry | letter |  |
| bubble | hurry | furry | valley | money | berry | leather | lever | banner |  |
| body | berry | ladder | dairy | candy | tackle | paddle | furry | puddle |  |
| meter | leather | money | babble | lever | candle | polar | muscle | cattle |  |
| shallow | battle | worry | paddle | letter | mayor | lighter | valley | tackle |  |
| **10 Words per List** | | | | | | | | | |
| candle | banner | valley | puddle | worry | muscle | meter | lighter | babble | shallow |
| ladder | muscle | meter | puddle | candy | lighter | bubble | tackle | polar | mayor |
| leather | valley | battle | tackle | shallow | ladder | meter | candle | bubble | muscle |
| hurry | candy | ladder | paddle | berry | banner | furry | meter | dairy | worry |
| letter | berry | mayor | bubble | cattle | money | candy | lever | muscle | polar |
