# Supplementary material for: An account of the Speech-to-Song Illusion using Node Structure Theory
Source: PLoS One. 2018 Jun 8;13(6):e0198656. doi: 10.1371/journal.pone.0198656 (PMC5993277; doi:10.1371/journal.pone.0198656)
Supplement: S6 Appendix — Lists vary in the number of words and the number of syllables from 1 to 6 words and 2 to 12 syllables. All word-lists are given. (DOCX) [file pone.0198656.s006.docx]

**S6 Appendix. Word-Lists used in Experiment 6.** Lists vary in the number of words and the number of syllables from 1 to 6 words and 2 to 12 syllables. All word-lists are given.

| **1 Word, 2 Syllables per List** | | | | | |
| --- | --- | --- | --- | --- | --- |
| derby |  |  |  |  |  |
| cocoon |  |  |  |  |  |
| essence |  |  |  |  |  |
| logic |  |  |  |  |  |
| throttle |  |  |  |  |  |
| **1 Word, 4 Syllables per List** | | | | | |
| anatomy |  |  |  |  |  |
| immediate |  |  |  |  |  |
| recreation |  |  |  |  |  |
| biography |  |  |  |  |  |
| ultimatum |  |  |  |  |  |
| **2 Words, 4 Syllables per List** | | | | | |
| elbow | brother |  |  |  |  |
| react | angry |  |  |  |  |
| humor | achieve |  |  |  |  |
| courage | measure |  |  |  |  |
| puppet | fatigue |  |  |  |  |
| **2 Words, 8 Syllables per List** | | | | | |
| voluntary | diameter |  |  |  |  |
| economy | monopoly |  |  |  |  |
| generalize | obligation |  |  |  |  |
| eventual | ordinary |  |  |  |  |
| secondary | initiate |  |  |  |  |
| **3** **Words, 6 Syllables per List** | | | | | |
| damage | fluid | observe |  |  |  |
| network | baton | devote |  |  |  |
| convey | purchase | luggage |  |  |  |
| wallet | empty | corrupt |  |  |  |
| moisture | junior | acid |  |  |  |
| **3 Words, 12 Syllables per List** | | | | | |
| supervision | academic | morality |  |  |  |
| alleviate | technology | phenomenon |  |  |  |
| colonial | publicity | dietary |  |  |  |
| reality | limitation | tolerable |  |  |  |
| mandatory | valuable | comedian |  |  |  |
| **4 Words, 8 Syllables per List** | | | | | |
| signal | ethnic | kitchen | awkward |  |  |
| bazaar | donate | napkin | review |  |  |
| dainty | violet | govern | chamber |  |  |
| blossom | creature | drama | option |  |  |
| improve | sincere | jagged | buffet |  |  |
| **6 Words, 12 Syllables per List** | | | | | |
| bundle | message | assume | lagoon | exit | organ |
| neutral | remove | bishop | cradle | sugar | joyous |
| unite | chimney | wagon | magic | admire | rodent |
| orchard | lecture | giant | impose | unique | crucial |
| furnace | routine | hygiene | alarm | cigar | voyage |
